# Genetic and Proteinic Linkage of MAO and COMT with Oral Potentially Malignant Disorders and Cancers of the Oral Cavity and Pharynx

**DOI:** 10.3390/cancers13133268

**Published:** 2021-06-29

**Authors:** Ping-Ho Chen, Yen-Yun Wang, Ting-Hsun Lan, Leong-Perng Chan, Shyng-Shiou Yuan

**Affiliations:** 1 School of Dentistry, College of Dental Medicine, Kaohsiung Medical University, Kaohsiung 807378, Taiwan; phchen@kmu.edu.tw (P.-H.C.); wyy@kmu.edu.tw (Y.-Y.W.); tinghsun.lan@gmail.com (T.-H.L.); 2 Institute of Biomedical Sciences, National Sun Yat-Sen University, No. 70 Lienhai Road, Kaohsiung 804201, Taiwan; 3 Department of Veterinary Medicine, College of Veterinary Medicine, National Pingtung University of Science and Technology, Pingtung 912301, Taiwan; 4 Center for Cancer Research, Kaohsiung Medical University, Kaohsiung 807378, Taiwan; 5 Cancer Center, Kaohsiung Medical University Hospital, Kaohsiung Medical University, Kaohsiung 807378, Taiwan; 6 Cohort Research Center, Kaohsiung Medical University, Kaohsiung 807378, Taiwan; oleon24@yahoo.com.tw; 7 Division of Prosthodontics, Department of Dentistry, Kaohsiung Medical University Hospital, Kaohsiung 807378, Taiwan; 8 Faculty of Medicine, College of Medicine, Kaohsiung Medical University, Kaohsiung 807378, Taiwan; 9 Department of Otorhinolaryngology-Head and Neck Surgery, Kaohsiung Municipal Ta-Tung Hospital and Kaohsiung Medical University Hospital, Kaohsiung 807378, Taiwan; 10 Department of Medical Research, Kaohsiung Medical University Hospital, Kaohsiung 807378, Taiwan; 11 Graduate Institute of Medicine, College of Medicine, Kaohsiung Medical University, Kaohsiung 807378, Taiwan; 12 Translational Research Center, Kaohsiung Medical University Hospital, Kaohsiung 807378, Taiwan; 13 Department of Obstetrics and Gynecology, Kaohsiung Medical University Hospital, Kaohsiung 807378, Taiwan

**Keywords:** betel quid, cancers of the oral cavity and pharynx, oral potentially malignant disorders, monoamine oxidase, catechol-O-methyltransferase

## Abstract

**Simple Summary:**

The prevention and treatment of cancers of the oral cavity and pharynx are currently important issues for national health. Currently, the incidence of oral cavity and pharynx cancers is globally the highest in Taiwanese men. Regarding the occurrence of oral cavity and pharynx cancers and oral potentially malignant disorders (OPMD), no report has ascertained how betel quid (BQ) can induce the expression of monoamine oxidase (MAO) and catechol-O-methyltransferase (COMT). We aimed to explore the role and clinical significance of specific markers of BQ exposure and human susceptibility to MAO and COMT. Our findings highlight the association of MAO and COMT biomarkers to risks of oral and pharyngeal cancers and OPMD. These novel findings will provide important strategies for disease prevention, early clinical diagnosis, and treatment effectiveness, and will offer a strong foundation to reduce BQ-related cancers of the oral cavity and pharynx and OPMD.

**Abstract:**

Betel quid (BQ), a group I human carcinogen, strongly contributes to an increased risk of oral potentially malignant disorders (OPMD) and cancers of the oral cavity and pharynx. This study was conducted to discover whether monoamine oxidase (MAO) and catechol-O-methyltransferase (COMT) variants play a potential role in the risk assessment of oral cavity and pharynx cancers and OPMD, particularly among BQ users. We applied a case–control study to confirm the polymorphism of MAO and COMT using single-nucleotide polymorphisms. We used qRT-PCR, Western blotting, and immunohistochemistry (IHC) to determine MAO and COMT expression. Carriers of the MAOA rs6323 G-allele, MAOB rs6324 G-allele, and COMT rs4633 C/C-genotype had a prominently increased risk of oral cavity and pharynx cancers (AOR = 56.99; *p* < 0.001). Compared to adjacent noncancerous tissues, a significant downregulation of MAO and COMT expression was exhibited in cancerous tissues (*p* < 0.01). Furthermore, in different cell models, MAO and COMT expression was significantly downregulated with an increased dose of arecoline (*p* < 0.01). In personalized preventive medicine for oral and pharyngeal cancers, our findings are the first to demonstrate the potential role of lower MAO and COMT expression levels, with the risk polymorphisms utilized as clinical biomarkers.

## 1. Introduction

Globally, oral and pharyngeal cancers rank as the seventh most common cancer and as the ninth most common cause of death from cancer [1]. Among Taiwanese men, the incidence of oral cavity and pharynx cancers is the highest in the world [2]. In 2018, the age-standardized incidence rate was 42.15/100,000 persons for oral and pharyngeal cancers in Taiwanese men [3]. In Taiwanese men, cancers of oral and pharyngeal cancers are the fourth most common cancers (a morality rate of 15.48/100,000) [3]. Oral and pharyngeal cancers are closely associated with alcohol, tobacco, and betel quid (BQ) consumption. In Taiwan, an elevated risk of oral cavity and pharynx cancers and oral potentially malignant disorders (OPMD) (for example oral leukoplakia and oral submucous fibrosis) were associated with the consumption of BQ [2].

After the consumption of caffeine, nicotine, and alcohol, BQ is the fourth most frequently consumed psychoactive substance and contains areca nut (AN), betel leaf, and slaked lime, with or without varied local flavorings [4]. Worldwide, it has been estimated that approximately 600 million chewers (10% of the world population) chew a variety of BQ, primarily in Southeast and South Asia, on the South Pacific islands, and among immigrants of South Asia in immigrant communities (such as Africa, Australia, the United States, and the United Kingdom) [2]. In Taiwan, there are above two million habitual users (10% of the population) [5]. BQ is most commonly used by men (males: 16.5%; females: 2.9%) [5], aborigines, blue-collar workers, those with lower education levels, cigarette smokers, and those who drink alcohol drink. An inter-country collaborative study also indicated that men had a prominently higher prevalence of chewing rates (15.6%) than women (3.0%) in Taiwan [6].

Physiologically active monoamine neurotransmitters, including dopamine, norepinephrine, and epinephrine, are known as catecholamines. Catecholamines are degraded into their metabolites either by monoamine oxidase (MAO), which is commonly found in the mitochondrial outer membrane of the cell and/or by catechol-O-methyltransferase (COMT) located within the cytosol of cell. MAO families, including MAOA and MAOB, are mitochondrial enzymes [7] and are located on chromosomes Xp11.3 and Xp11.23, respectively [8]. MAO families can catalyze the oxidative deamination of monoamine neurotransmitters (such as dopamine, serotonin, norepinephrine, and epinephrine) to stimulus motor, memory, mood, and addictive behaviors [9,10], and catalyze the deamination of biogenic amines (tyramine) in the diet. MAOA is an important determinant of the activity of MAO [11]; the MAOB is related to dopamine and phenylethylamine metabolism [11]. In neurological diseases, MAO’s function is well established, but the role of MAO in carcinogenesis seems to be different [12]. MAOA was originally recognized as a regulator of neurotransmitters, but recent reports have shown that MAOA has unexpected roles in tumorigenesis [13,14]. Indeed, in various types of human cancers, a previous report demonstrated the downregulation of MAOA [13].

COMT is located on chromosome 22q11.2 and includes six exons [15]. COMT is an enzyme involved in the degradation of catecholamines (such as dopamine, norepinephrine, and epinephrine) metabolism and is commonly known to catalyze catechol estrogens that are potentially carcinogenic and have a DNA-damaging ability [16]. The role of COMT may be implicated in cancer and neurological and cardiovascular disorders [17]. Nevertheless, less attention has been paid to the possible contribution of COMT to the development of cancers.

Arecoline is the main ingredient in the alkaloids of areca nut. Our previous study has hinted that specific MAOA genetic polymorphism is associated with heavy BQ users [18]. In the brain, MAO and COMT are enzymes involved in the degradation of neurotransmitters and their inhibitors may deter the breakdown of neurotransmitters and are used for treatment of neurodegenerative diseases such as Parkinson’s disease. Since arecoline plays a role in the inhibition of MAOA and is responsible for BQ dependence [19], we speculate that MAO and COMT may be involved in the development of oral and pharyngeal cancers.

MAO and COMT were originally recognized as regulators of neurotransmitters, but recent reports have discovered that they have unexpected roles in tumorigenesis. To the best of our knowledge, the joint effects of associations between MAO and COMT variants on the risks of oral cavity and pharynx cancers and OPMD have not been reported. We assume that three biomarker (MAOA, MAOB, and COMT) variants may contribute to oral and pharyngeal cancers occurrence and may be implicated in the induction of arecoline. Therefore, this study was conducted to discover whether MAO and COMT variants contribute a potential role in the risk assessment of oral and pharyngeal cancers and OPMD, particularly among BQ chewers.

## 2. Materials and Methods

### 2.1. Study Subjects

A case–control study recruited 530 male subjects, composed of 297 male patients with oral cavity and pharynx cancers, 40 male patients with OPMD, and 193 healthy male controls. This Institutional Review Board (IRB) of clinical study was permitted from Kaohsiung Medical University (KMU) Hospital (KMUHIRB-G(I)-20160014). Male patients with oral cavity and pharynx cancers were recruited from the KMU hospital. We explained the purpose of this study and all procedures to all participants in this study. All subjects agreed to sign a written informed consent form for this study, and it included all participants who accepted to response the questionnaire by trained interviewers and agreed to provide the blood and specimens for experimental analysis. The questionnaire data included personal demographic data, previous exposure history of substance use (particularly betel use), and clinical characteristics. Subjects with oral and pharyngeal cancers voluntarily agreed to offer oral and pharyngeal cancerous tissue and adjacent non-cancerous oral tissue.

The minimum sample size of tissue was calculated by G Power software (version 3.1.9.4). In order to achieve 85% statistical power for this study (an effect size of 0.50 and a 0.05 type I (α) error), the estimation of sample size was that at least 40 participants should be recruited. In order to exclude the individual differences of mRNA and protein expressions of MAO and COMT, a total of 46 cancerous tissues and their paired non-cancerous tissues were collected. Subsequently, we excluded 4 female specimens, and, finally, we collected 42 eligible subjects. Of note, all our subjects (*n* = 42) for evaluating the mRNA and protein expressions were from the same participants of the large case–control study. All oral and pharyngeal cancers or OPMD cases without radiation therapy or chemotherapy were histologically confirmed by clinical surgeons or pathologists. These specimens were collected for gene expression and SNP assays of MAO and COMT.

### 2.2. Genetic Polymorphisms Analysis of MAO and COMT

Single-nucleotide polymorphisms (SNPs) of MAOA (rs6323, rs1137070, and rs5906957), MAOB (rs6324, rs1799836, and rs3027452), and COMT (rs4633, rs9605030, rs9606186) were chosen with the frequency of minor alleles > 0.1 in the Chinese HapMap-CHB (a public reference database) and Haploview (version 4.2). According to the instructions of manufacturer, the genotyping of SNPs was analyzed with a TaqMan genotyping assay. All assays and gDNA, as well as PCR, were conducted in 384-well plates. After using amplification of PCR, an endpoint plate read was performed with a Real-Time PCR System (Applied Biosystems ViiA 7).

### 2.3. Single Nucleotide Polymorphism Assay

QPCR SNP dual fluorescent labeled probe assays for rs6323, rs1137070, rs5906957, rs6324, rs1799836, rs3027452, rs4633, rs9605030 and rs9606186 were custom designed by Topgen Biotechnology (Topgen Biotech., Kaohsiung, Taiwan). Sequence and labeled dye information of primer and probes as listed in Table S1. Briefly, QPCR SNP performed with 2X AceGT Genotyping Master Mix (Topgen Biotech., Kaohsiung Taiwan) on ViiA™ 7 Real Time PCR (Applied Biosystems, Waltham, MA, USA). QPCR program is 95 °C 5 min, 60 °C 30 s with data collection for pre-reading, 40 cycles of 95 °C 3 s and 60 °C 40 s with data collection each cycle end at 60 °C, final step is 60 °C 30 s with data collection for post-reading. Allelic discrimination plots were analyzed by ViiA™ 7 SW v1.3.

### 2.4. Cell Cultures and Cytotoxicity Assay

In this study, two human oral cancer cell lines (OECM-1 and HSC-3), human dysplastic oral keratinocyte (DOK) representing OPMD cells and normal human oral keratinocytes (HOK), were incubated at 37 °C in a 5% CO_2_ incubator. The human oral squamous carcinoma cell line OECM-1 (from a Taiwanese betel quid chewer) and HSC-3 tongue squamous carcinoma cell line are well-established models for squamous cell carcinoma [20,21]. OECM-1 and HSC-3 cells were grown in Dulbecco’s modified Eagle’s medium (DMEM). DOK cells were incubated in high glucose DMEM medium supplemented with 10% fetal bovine serum (HyClone, Logan, UT, USA), 2 mM glutamine (HyClone, Logan, UT, USA), 5 µg/mL hydrocortisone (HyClone, Logan, UT, USA), 100 µg/mL streptomycin (HyClone, Logan, UT, USA), and 100 U/mL penicillin–streptomycin (Invitrogen, Carlsbad, CA, USA). Normal HOK cells (ScienCell, Carlsbad, USA) were incubated in oral keratinocyte medium (OKM, Cat. #2611, ScienCell, Carlsbad, USA). The medium of cell culture was refreshed every three days. Detailed information on cell culture has been reported in our previous study [20,21].

We added different concentrations (0, 20, 40, 60, 80, and 100 µM) of arecoline to HOK for 24 h to assess cell viability (Figure S1). We added MTT solution (5 mg/mL) to the cells and incubated them for 2 h at 37 °C in a CO_2_ incubator. Subsequently, the viable cell percentage compared with vehicle controls was calculated by using an ELISA reader (el800, Bio Tek, Winooski, VT, USA).

### 2.5. Real-Time qRT-PCR Analysis

We used the RNA Extraction Kit to extract total RNA of cells or tissues. The sample RNA concentration was quantified using a NanoDrop spectrophotometer (MEDCLUB, Nano-200, Taiwan.). We used 1 μg RNA for synthesis of first-strand complementary (c) DNA by a TOOLS Easy Fast RT Kit according to the manufacturer’s instructions (TOOLS Biotech, Taiwan, Cat. No. KRT-BA06-2). qRT-PCR was conducted using TOOLS 2X SYBR qPCR Mix (TOOLS Biotech, Taiwan, Cat. No. FPT-BB05-10) and StepOne™ System (Thermo Scientific, USA). Amplifications were calculated and normalized to GAPDH with the 2^−^^△△Ct^ method [21]. Compared to the GAPDH gene (internal control), the expression the target gene was calculated using the formula: 2^−^^△△Ct^, where △Ct = Ct target gene − Ct internal control, and △△Ct = △Ct test sample − △Ct control sample in each sample. Relative quantification using the formula 2^−∆∆Ct^ was used to calculate the average of fold change with standard deviation (SD) for triplicate determinations [21]. The detail information of MAO/COMT primers used in qRT-PCR is shown in Table S2.

### 2.6. Protein Extraction and Western Blotting

Separated proteins were transferred to polyvinylidene fluoride membranes subsequently. The membranes were incubated with primary antibodies (MAOA (ab126751, Abcam) (1:200), MAOB (GTX105970, GeneTex) (1:200), COMT (D4N6M#14368) (Cell Signaling Technology) (1:100), and β-actin (A5316, MilliporeSigma, St. Louis, MO, USA) for 2 h at room temperature, after incubation for 1 h in the blocking buffer. An imaging system of MiniChemiTM and detection system were used to detect specific protein bands.

### 2.7. Immunohistochemistry

The antibodies used were as follows: MAOA (ab126751, Abcam) (1:200), MAOB (ab133270, Abcam) (1:200), COMT (D4N6M#14368) (Cell Signaling Technology) (1:100), and β-actin (A5316, Sigma). Notably, only staining of tumor cells was calculated. Briefly, each slide of immunohistochemical (IHC) staining for the scoring system was used to classify the samples into intensity categories: a low expression level and a high expression level. The following scale was used to present the intensity of staining of the tumor stroma: no (staining score = 0), weak (staining score = 1), moderate (staining score = 2), and strong (staining score = 3). Another score showing the proportion of positively stained tumor cells was graded as follows: <10% positive tumor cells (proportion score = 0), 11–25% (proportion score = 1), 26–50% (proportion score = 2), 51–75% (proportion score = 3), and >75% (proportion score = 4). Finally, the IHC scores were multiplied by each of the two scales to obtain a composite value. A Tekfar digital eyepiece camera 5.0 MP light microscope and Rising view version 3.7 was used to capture the representative field photographs.

### 2.8. Statistical Analysis

We applied mean ± standard deviation (SD) or proportion (%) to depict distribution of subject demographic factors and the quantity and time of substance use (alcohol, BQ, and cigarette). After cells were treated with different concentrations, the general linear model (GLM) analysis was applied for multiple comparisons and conducted to analyze the mean differences of MAO and COMT expression in cell models or the independent *t*-test for two independent sample comparisons. As the mRNA and protein expression values showed a non-normal distribution, the Wilcoxon signed-rank non-parametric test was applied to calculate the expression levels of MAO and COMT in oral cavity and pharynx cancer tissues when compared with their adjacent non-cancerous tissues. In addition, the Mann–Whitney U test was used for two independent sample comparisons.

The curves of characteristic (ROC) were determined to distinguish the mRNA levels of MAO and COMT. The Hardy–Weinberg equilibrium was used to compare the proportion of observed and expected genotypes by using the χ^2^ test to evaluate deviation from the cases and controls, respectively. The Chi-square (χ^2^) test and an unconditional logistic regression model were applied to estimate the distribution of demographic factors and their association between diseases and controls. An unconditional multiple logistic regression model controlling for potential confounding factors was used to determine adjusted odds ratio (AOR), 95% confidence interval (CI), and exact *p* values. As stated above, the statistical methods followed the reported methods from our previous works [22,23]. The Spearman correlation coefficient was used to calculate the associations between the three biomarker mRNA values and cumulative lifetime BQ use (pack-years). If a Spearman’s correlation (ρ) is 0.00 ≤ ρ < 0.20, the research findings are considered no correlation. If a Spearman’s correlation is 0.21 ≤ ρ < 0.40, the research findings are considered low correlation. If a Spearman’s correlation is 0.41 ≤ ρ < 0.60, the research findings are considered moderate correlation. If a Spearman’s correlation is 0.61 ≤ ρ < 0.80, the research findings are considered marked correlation. If a Spearman’s correlation is 0.81 ≤ ρ < 1.00, then the research findings are considered high correlation [24,25].

An age-matched analysis was conducted in this study. We used propensity-score matching to ascertain case–control participants with similar baseline age. Age matching was analyzed with a 1:1 matching protocol without replacement (greedy-matching algorithm), and this calculation was with a caliper width equal to 0.25 of standard deviation of logit propensity score. All the statistical analyses were performed using the SPSS version 20 of the SPSS Statistical Package (SPSS Institute Inc., Chicago, DE, USA), version 9.4 of SAS Statistical Package (SAS Institute Inc., Cary, NC, USA), and version 20 of the MedCalc software (Mariakerke, Belgium: MedCalc Software Ltd., 2021), which was applied for statistical analysis and graphical representation of the data. The results indicated by asterisks are considered statistically significant with the two-tailed test (*p* < 0.05*, and < 0.01**).

## 3. Results

### 3.1. Characterization of Study Population

A total of 530 participants were enlisted in this case–control study. They were divided into three groups: 297 men with oral cavity and pharynx cancers, 40 men with OPMD, and 193 healthy controls. Table 1 denotes the characteristics of sociodemographic variables, alcohol drinking, BQ chewing, and cigarette consumption in the patients and healthy controls. The average age of diagnosis was 53.97 ± 10.32 years old in oral and pharyngeal cancers, 50.83 ± 11.57 years old in OPMD, and 46.08 ± 12.94 years old in controls, and the average age of patients with oral cavity and pharynx cancers was older than controls significantly (*p* < 0.001). A total of 81.9% of the healthy controls were of Minnan ethnicity, 74.1% of the oral and pharyngeal cancer patients were of Minnan ethnicity, and 62.5% of the OPMD patients were of Minnan ethnicity. A significantly higher education level was found in controls than in patients with oral and pharyngeal cancers and OPMD (*p* < 0.001). The current or former use of alcohol, BQ, and cigarettes was significantly more prevalent in the oral and pharyngeal cancer and OPMD patients than in the healthy controls (*p* < 0.01). The cumulative lifetime BQ use (pack-years) was 76.10 ± 89.07 in BQ chewers with oral cavity and pharynx cancers, which is higher than those of healthy BQ chewers (41.56 ± 37.10) significantly. The proportion of BQ juice swallowing was significantly higher in oral and pharyngeal cancer patients (42.4%) and OPMD (45.5%) than in healthy controls (14.3%). Oral and pharyngeal cancer patients were significantly older (45.45 ± 11.49) at the time of quitting of chewing compared to healthy controls (36.84 ± 9.76).

### 3.2. SNPs of MAO/COMT and Substance Use (BQ, Cigarette, and Alcohol) on Risk Assessment of Oral and Pharyngeal Cancers and OPMD

The risk assessment of MAO and COMT polymorphisms frequency and BQ use habits (Yes: +; No: −) among oral cavity and pharynx cancers, OPMD, and healthy controls are shown in Table 2. After adjusting for covariates, it was observed that those with the MAOA (rs6323) risk G-allele were significantly related to the risks of oral cavity and pharynx cancers (AOR = 1.98, 95% CI = 1.23–3.20) and OPMD (AOR = 2.89, 95% CI = 1.27–6.58), compared to the healthy control group (MAOA (rs6323) T-allele). Subjects with the MAOA (rs1137070) risk T-allele were significantly associated with the risks of oral cavity and pharynx cancers (AOR = 2.37, 95% CI = 1.47–3.81) and OPMD (AOR = 3.25, 95% CI = 1.43–7.38). The MAOA (rs5906957) risk A-allele was significantly associated with the risks of oral and pharyngeal cancers (AOR = 1.67, 95% CI = 1.03–2.71) and OPMD (AOR = 2.55, 95% CI = 1.11–5.85). In particular, we found that BQ chewers with the MAOA (rs6323) risk G-allele had a significantly synergistic risk of oral and pharyngeal cancers (AOR = 31.15; 95% CI, 13.43–72.27) and OPMD risk (AOR = 12.77; 95% CI, 3.61–45.20) compared to non-BQ chewers with MAOA rs6323 (T-allele). Likewise, BQ chewers with MAOA rs1137070 (T-allele) had a significantly synergistic risk of oral and pharyngeal cancers (AOR = 36.22; 95% CI, 16.24–80.78) and risk of OPMD (AOR = 15.47; 95% CI, 4.67–51.26). Compared to non-BQ chewers with MAOA rs5906957 (G-allele), BQ chewers with MAOA rs5906957 (A-allele) had a significantly synergistic risk of oral and pharyngeal cancers (AOR = 24.15; 95% CI, 10.44–55.89) and risk of OPMD (AOR = 10.04; 95% CI, 2.83–35.56).

The risk assessment of MAOB frequency among oral cavity and pharynx cancers, OPMD, and healthy controls is shown in Table 2. Compared to the healthy control group (MAOB (rs6324) A-allele), the MAOB (rs6324) risk G-allele was significantly related to the risks of oral cavity and pharynx cancers (AOR = 13.00, 95% CI = 7.35–22.98) and OPMD (AOR = 37.45, 95% CI = 11.94–117.46). We found that BQ chewers with MAOB (rs6324) risk G-allele had a significantly synergistic risk of oral and pharyngeal cancers (AOR = 307.03; 95% CI, 97.43–967.54). Likewise, BQ chewers with MAOB rs1799836 (T-allele) had a prominently synergistic risk of oral and pharyngeal cancers (AOR = 22.60; 95% CI, 7.13–71.62) and risk of OPMD (AOR = 6.32; 95% CI, 1.21–33.08). Compared with non-BQ chewers with MAOA rs3027452 (A-allele), BQ chewers with MAOA rs3027452 (G-allele) had a significantly synergistic risk of oral and pharyngeal cancers (AOR = 18.57; 95% CI, 5.06–68.18).

The risk assessment of COMT genotype frequency variants among oral and pharyngeal cancers, OPMD, and healthy controls is shown in Table 2. After adjusting for covariates, compared to the healthy control group with the COMT (rs4633) T/T+C/T combined genotype as the reference group, the COMT (rs4633) risk C/C was significantly related to the risks of oral cavity and pharynx cancers (AOR = 1.78, 95% CI = 1.11–2.85) and the risk of OPMD (AOR = 2.65, 95% CI = 1.19–5.88). In particular, BQ chewers with the at-risk rs4633 C/C genotype had a significantly synergistic risk of oral and pharyngeal cancers (AOR = 27.14; 95% CI, 12.16–60.56) and risk of OPMD (AOR = 26.86, 95% CI = 5.24–137.65). Compared to the healthy control group with rs9605030 C/C+C/T, BQ chewers with the COMT (rs9605030) risk T/T genotype had a significantly synergistic risk of oral and pharyngeal cancers (AOR = 31.57; 95% CI, 6.10–163.34) and risk of OPMD (AOR = 32.29, 95% CI = 3.28–317.97). Compared to the healthy control group (COMT (rs9606186) C/C+G/C genotype), BQ chewers with the COMT rs9606186) risk G/G was significantly related to the risks of oral cavity and pharynx cancers (AOR = 22.13, 95% CI = 10.23–47.84), and the risk of OPMD (AOR = 20.73, 95% CI = 4.97–86.52).

To confirm the differences in the distribution of MAOA, MAOB, and COMT among male patients with oral cancer (*n* = 209), pharyngeal cancer (*n* = 88), OPMD (*n* = 40), and control (*n* = 193), we divided the patients into four groups. A similarly significant pattern was confirmed in the distribution of candidate genotypes of MAOA, MAOB, and COMT among men with oral cancer (*n* = 209), pharyngeal cancer (*n* = 88), and OPMD (*n* = 40), and controls (*n* = 193) (Table S3). Compared to healthy controls, patients with risk polymorphisms of MAOA, MAOB, and COMT had a significantly enhanced risk of oral cancer. A significantly elevated risk of OPMD was also found in patients with risk polymorphisms of MAOA, MAOB, and COMT compared to healthy controls (Table S3). To explore whether smoking or drinking habits showed synergistic effects for the risk of oral and pharyngeal cancer in subjects with higher susceptibility SNPs of MAO/COMT, the synergistic effects of cigarette smoking, alcohol drinking, and higher susceptibility SNPs of MAO/COMT on oral and pharyngeal cancers and OPMD were calculated by stratifying the uses of cigarette and alcohol across the susceptibility SNPs of MAO/COMT (Tables S4 and S5). There were non-significant or lower synergistic effects for smokers combined with the susceptibility SNPs of MAO/COMT on most of the OR ratios between oral and pharyngeal cancers and OPMD (Table S4). Similarly, non-significant risk patterns were observed among alcohol drinkers with oral and pharyngeal cancers or OPMD (Table S5). These results reveal that smokers or drinkers with risk SNPs of MAO/COMT showed non-significant or lower synergistic effects on the risks of oral and pharyngeal cancers or OPMD. Conversely, the synergistic effects were very prominent among BQ chewers with risk polymorphisms of MAO/COMT.

As a significant age difference was present between oral and pharyngeal cancers (53.97 ± 10.32) and control groups (46.08 ± 12.94), we conducted age-matched analysis to confirm the difference of odds ratios (OR) before and after age-matching. Distribution of significantly demographic characteristics and substance use among males with oral and pharyngeal cancers and control were calculated after propensity-score matching for age (Table S6). Due to the limited size of the control group, we only matched 150 cases: 150 controls with a 1:1 age matching protocol. In Table S7, after using age-matching analysis, the results showed similar significances between pre-match for MAOA rs6323 G-allele (AOR = 1.98), MAOB rs6324 G-allele (AOR = 13.00); COMT rs4633 C/C (AOR = 1.78) and post-match for MAOA rs6323 G-allele (AOR = 2.13), MAOB rs6324 G-allele (AOR = 15.54); COMT rs4633 C/C (AOR = 2.12), respectively.

### 3.3. Gene–Gene Joint Effects of MAO/COMT SNPs on Risk Assessment

The joint effects of MAO/COMT SNPs and BQ use on risk assessment among oral cavity and pharynx cancers, OPMD, and healthy controls are shown in Table 3. Although nine SNP sites are examined in Table 2, we selected four significant SNPs to examine the joint effects of MAO/COMT (Table 3) for their statistically significant *p* value ≤ 0.01 in MAOA/COMT SNP (rs6323 G-allele, *p* = 0.005; rs1137070 T-allele, *p* < 0.001; rs6324 G-allele, *p* < 0.001; rs4633 C/C genotype, *p* = 0.016). In terms of MAOA and MAOB, after adjusting for covariates, compared with the healthy control group (MAOA rs6323 T allele and MAOB rs6324 A allele), we found that subjects with MAOA rs6323 (G-allele) and MAOB rs6324 (G-allele) had a significantly increased risk of oral and pharyngeal cancers (AOR = 30.32; 95% CI, 13.07–70.35) and risk of OPMD (AOR = 53.21; 95% CI, 10.91–259.62). Subjects with MAOA rs1137070 (T-allele) and MAOB rs6324 (G-allele) had a significantly increased risk of oral and pharyngeal cancers (AOR = 45.56; 95% CI, 18.51–112.17) and risk of OPMD (AOR = 70.59; 95% CI, 14.17–351.78).

In terms of MAOA and COMT, subjects with MAOA rs6323 (G-allele) and COMT rs4633 (C/C genotype) had a significantly increased risk of oral and pharyngeal cancers (AOR = 2.94; 95% CI, 1.50–5.76) and risk of OPMD (AOR = 4.74; 95% CI, 1.54–214.57). Subjects with MAOA rs1137070 (T-allele) and COMT rs4633 (C/C genotype) had a significantly increased risk of oral and pharyngeal cancers (AOR = 3.57; 95% CI, 1.86–6.82) and risk of OPMD (AOR = 5.31; 95% CI, 1.76–16.00). In terms of MAOB and COMT, subjects with MAOB rs6324 (G-allele) and COMT rs4633 (C/C genotype) had a significantly increased risk of oral and pharyngeal cancers (AOR = 39.27; 95% CI, 15.66–98.47).

In terms of MAOA, MAOB, and COMT, subjects with the MAOA rs6323 G-allele, MAOB rs6324 G-allele, and COMT rs4633 C/C-genotype had a significantly increased risk of oral and pharyngeal cancers (AOR = 56.99; 95% CI, 15.82–205.31). Subjects with the MAOA rs1137070 T-allele, MAOB rs6324 G-allele, and COMT rs4633 C/C-genotype had a significantly increased risk of oral and pharyngeal cancers (AOR = 78.62; 95% CI, 20.37–303.45).

### 3.4. The mRNA and Protein Levels of MAO/COMT in Oral and Pharyngeal Cancerous Tissues and Non-Cancerous Tissues

We used oral and pharyngeal cancerous tissues and their paired tissues (adjacent non-cancerous tissues) from 42 patients to confirm mRNA and protein expression of MAO/COMT. These patients were compared for the distribution of selected demographic characteristics (Table S8). All the selected demographic characteristics were not different (*p* > 0.05) between the males with oral cancer and pharyngeal cancer.

**Figure 1:**
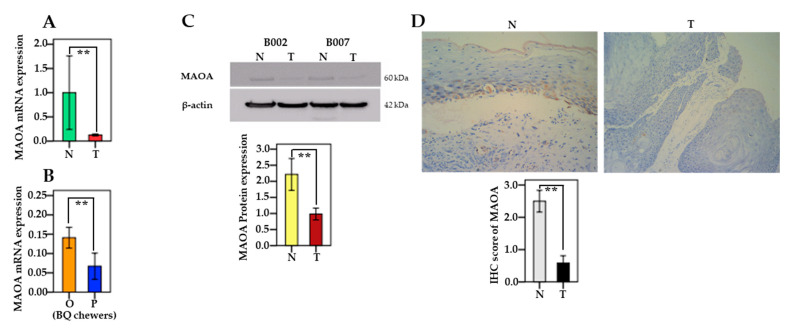
MAOA expression in oral and pharyngeal cancer patients. (**A**) Differences in the expression of MAOA mRNA between tumor (T) tissue and adjacent normal tissue (N). The relative expression (2^−^^△△Ct^) of MAOA mRNA was normalized to that of GAPDH. (*n* = 42; *p* = 0.001). (**B**) Relationship between MAOA mRNA expression and cancerous tumor sites (oral (O) vs. pharynx (P)) in BQ chewers (*n* = 37; *p* = 0.009). (**C**) Level of MAOA protein expression in tumor (T) tissues compared with adjacent normal (N) tissues presented by relative density (target gene/β-actin) (*n* = 20; *p* = 0.006) (two random patients are shown, and β-actin was used as a control for protein loading). (**D**) The protein expression of MAOA between tumor tissues and adjacent non-cancerous tissues was analyzed by immunohistochemistry (IHC) (*n* = 12; *p* = 0.006). The representative IHC images have a x200 magnification of MAOA expression in tumor tissue and adjacent non-cancerous tissue. The data were summarized from at least three independent experiments. Bars are shown as mean ± SEM. Significant differences were analyzed with the paired Wilcoxon-signed ranks test or the independent Mann–Whitney U test. ** *p* < 0.01.

Compared to adjacent non-cancerous tissues, MAOA mRNA levels was significantly downregulated in oral and pharyngeal cancerous tissues (*n* = 42; *p* = 0.001) (Figure 1A). In BQ chewers, lower expression of MAOA mRNA was found in the pharyngeal site than in the oral sites (*p* = 0.009) (Figure 1B). Furthermore, using Western blotting, we examined the quantitative protein expression of MAOA in 20 patients and found that MAOA was significantly downregulated in cancerous oral cavity and pharynx tissues compared to that in adjacent non-cancerous tissues (*p* = 0.006) (Figure 1C). Furthermore, representative IHC images of MAOA expression in tumor tissue and adjacent non-cancerous tissue are shown in Figure 1D. When we used the IHC scores to confirm MAOA protein expression, our results showed that the scores in cancerous tissues were lower than those in adjacent non-cancerous tissues significantly (*n* = 12; *p* = 0.006).

**Figure 2:**
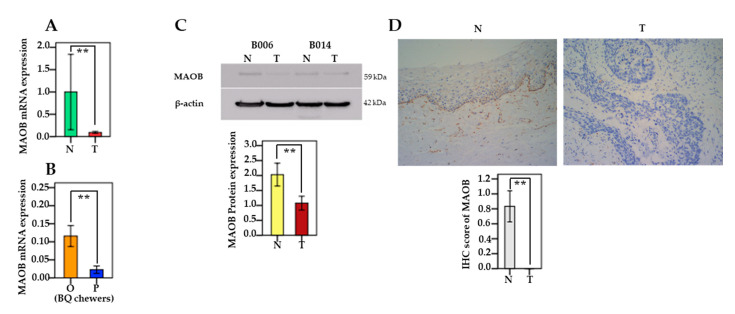
MAOB expression in oral and pharyngeal cancer. (**A**) Differences in the expression of MAOB mRNA between tumor (T) tissue and adjacent normal tissue (N). The relative expression (2^−^^△△Ct^) of MAOB mRNA was normalized to that of GAPDH. (*n* = 42; *p* = 0.004). (**B**) Relationship between MAOB mRNA expression and cancerous tumor sites (oral (O) vs. pharynx (P)) in BQ chewers (*n* = 37; *p* = 0.006). (**C**) Level of MAOB protein expression in tumor (T) tissues compared with adjacent normal (N) tissues presented by relative density (target gene/β-actin) (*n* = 20; *p* = 0.002) (two random patients are shown, and β-actin was used as a control for protein loading). (**D**) The protein expression of MAOB between tumor tissues and adjacent non-cancerous tissues was analyzed by immunohistochemistry (IHC) (*n* = 12; *p* = 0.008). Representative IHC images have a x200 magnification of MAOB expression in tumor tissue and adjacent non-cancerous tissue. The data were summarized from at least three independent experiments. Bars are shown as mean ± SEM. Significant differences were analyzed with the paired Wilcoxon-signed ranks test or the independent Mann–Whitney U test. ** *p* < 0.01.

MAOB mRNA expression was significantly downregulated in the oral and pharyngeal cancerous tissues compared to the adjacent non-cancerous tissues (*n* = 42; *p* = 0.004) (Figure 2A). We also found lower expression of MAOA mRNA expression in the pharyngeal site compared to oral sites (*p* = 0.006) among BQ chewers. (Figure 2B). In addition, using Western blotting, we examined quantitative protein expression of MAOB in 20 patients and found that MAOB was significantly downregulated in oral and pharyngeal cancerous tissues compared to non-cancerous tissues (*p* = 0.002) (Figure 2C). We used IHC scores to confirm MAOB protein expression, and our results show that the scores were decreased in cancerous tissues significantly more than in adjacent non-cancerous tissues (*n* = 12; *p* = 0.008) (Figure 2D).

**Figure 3:**
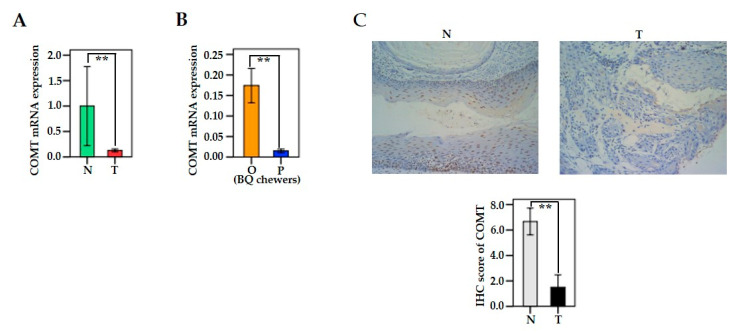
COMT expression in oral and pharyngeal cancer patients. (**A**) Differences in the expression of COMT mRNA between tumor (T) tissue and adjacent normal tissue (N). The relative expression (2^−^^△△Ct^) of COMT mRNA was normalized to that of GAPDH. (*n* = 42; *p* = 0.009). (**B**) Relationship between COMT mRNA expression and cancerous tumor sites (oral (O) vs. pharynx (P)) in BQ chewers (*n* = 37; *p* = 0.001). (**C**) The protein expression of COMT between tumor tissues and adjacent non-cancerous tissues was analyzed by immunohistochemistry (IHC) (*n* = 12; *p* = 0.007). Representative IHC images have a x200 magnification of COMT expression in tumor tissue and adjacent non-cancerous tissue. The data were summarized from at least three independent experiments. Bars are shown as mean ± SEM. Significant differences were analyzed with the paired Wilcoxon-signed ranks test or the independent Mann–Whitney U test. **p < 0.01.

COMT mRNA expression was significantly lower in oral and pharyngeal cancerous tissues than in adjacent non-cancerous tissues (*n* = 42; *p* = 0.009) (Figure 3A). In BQ chewers, COMT mRNA expression was significantly lower in pharyngeal tumor tissues than in oral cancerous tissues (*p* = 0.001) (Figure 3B). IHC analysis of oral and pharyngeal cancer cases indicated that COMT expression was significantly decreased in cancerous tissues compared to that in non-cancerous tissues (*n* = 12; *p* = 0.007) (Figure 3C).

### 3.5. Associations Between MAO/COMT mRNAs and Genetic Polymorphisms

**Figure 4:**
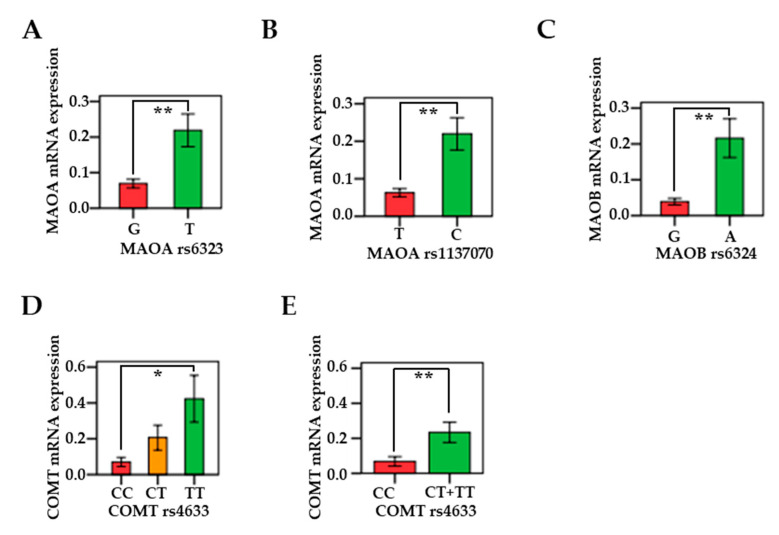
MAO/COMT mRNA expression in oral and pharyngeal cancer patients based on MAOA rs6323 G-allele, MAOB rs6324 G-allele, and COMT rs4633 C/C genotypes. (**A**) Differences in the expression of MAOA mRNA between rs6323 G (*n* = 26) and T (*n* = 16) alleles (*p* = 0.008). (**B**) Differences in the expression of MAOA mRNA between rs1137070 T (*n* = 25) and C (*n* = 17) alleles (*p* = 0.003). (**C**) Differences in the expression of MAOB mRNA between rs6324 G (*n* = 29) and A (*n* = 13) alleles (*p* < 0.001). (**D**) Differences in the expression of COMT mRNA between rs4633 CC (*n* = 27), CT (*n* = 12) and TT (*n* = 3) genotypes (*p* = 0.028). (**E**) Differences in the expression of COMT mRNA between rs4633 CC (*n* = 27) and CT + TT (*n* = 15) and TT (*n* = 3) genotypes (*p* = 0.006). * *p* < 0.05; **p < 0.01.

The MAOA rs6323 G-allele, MAOB rs6324 G-allele, and COMT rs4633 C/C genotypes were linked to decreased mRNA expression in oral and pharyngeal cancerous tissues. In Figure 4, the mRNA expression levels for the rs6323 G-allele (*n* = 26) in MAOA were significantly lower than the T-allele (*n* = 16) mRNA expression levels (*p* = 0.008) (Figure 4A). The mRNA expression levels for the rs1137070 T-allele (*n* = 25) in MAOA were significantly lower than the C-allele (*n* = 17) mRNA expression levels (*p* = 0.003) (Figure 4B). The mRNA expression levels for the rs6324 G-allele (*n* = 29) in MAOB were significantly lower than the A-allele (*n* = 13) mRNA expression levels (*p* < 0.001) (Figure 4C). In the COMT gene, the expression levels for the rs4633 C/C genotypes (*n* = 27) were lower compared to the C/T (*n* = 12) and T/T genotypes (*n* = 3). After post hoc comparison, the statistic was only significant between the C/C and TT genotypes (*p* = 0.028), showing a relationship between the levels of COMT mRNA and SNP polymorphism (Figure 4D). Additionally, the risk C/C genotype showed significantly lower mRNA expression compared to COMT (rs4633) C/T+C/C (*p* = 0.006) (Figure 4E).

Significantly strong positive associations were also found between the mRNAs of MAOA, MAOB, and COMT in the same cancer tissues (Figure S2; Table S9). The Spearman correlation coefficient (ρ) was 0.822 between the mRNAs of MAOA and MAOB in the same cancer tissues, indicating a significant positive association (*p* < 0.001). Moreover, increased expression of MAOB mRNA was significantly associated with COMT mRNA in the same cancer tissues (ρ = 0.738; *p* < 0.001), and increased expression of MAOA mRNA was significantly associated with COMT mRNA in the same cancer tissues (ρ = 0.557; *p* < 0.001).

### 3.6. Diagnostic Performance of MAO/COMT Biomarkers

**Figure 5:**
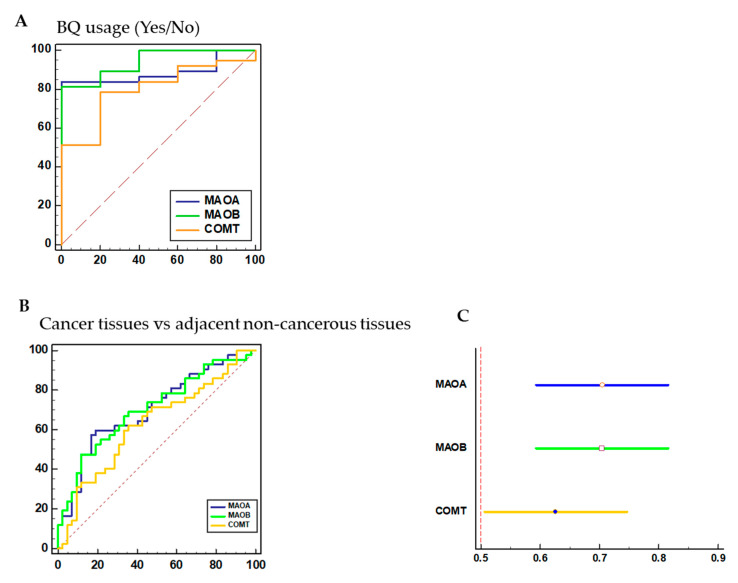
The ROC analysis was applied to the MAO/COMT biomarkers. (**A**) The AUC analysis of BQ usage (Yes/No) in the evaluation of discriminating power of mRNA expression of MAOA (AUC = 0.89; 95% CI = 0.75–0.96), MAOB (AUC = 0.94; 95% CI = 0.82–0.99), and COMT (AUC = 0.80; 95% CI = 0.65–0.91). (**B**) ROC and areas under the curve (AUC) for mRNA expression of MAOA, MAOB, and COMT in oral and pharyngeal cancer tissues compared with adjacent non-cancerous tissue (*n* = 42). (**C**) MAOA (AUC = 0.71; 95% CI = 0.59–0.82), MAOB (AUC = 0.70; 95% CI = 0.59–0.82) and COMT (AUC = 0.63; 95% CI = 0.51–0.75), respectively. AUC: area under the curve.

The AUC of MAO/COMT is significantly better for BQ use. In the AUC analysis of BQ usage (Yes/No), it was valuable for being a reference in the evaluation of discriminating power of mRNA expression of MAOA (AUC = 0.89; 95% CI = 0.75–0.96), MAOB (AUC = 0.94; 95% CI = 0.82–0.99), and COMT (AUC = 0.80; 95% CI = 0.65–0.91) (Figure 5A). The ROC and areas under the curve (AUC) analyses were applied to the MAO/COMT biomarkers in cancerous tissues compared with adjacent non-cancerous tissues (Figure 5B). The AUC for MAOA was 0.71 (95% CI = 0.59–0.82; *p* < 0.001), 0.70 (95% CI = 0.59–0.82; *p* < 0.001) for MAOB, and 0.63 (95% CI = 0.51–0.75; *p* = 0.0420) for COMT, respectively (Figure 5C). Accordingly, these results suggest that MAOA, MAOB, and COMT mRNA biomarkers may significantly discriminate between cancerous and non-cancerous tissues.

### 3.7. Associations between MAO/COMT mRNA and BQ Use

**Figure 6:**
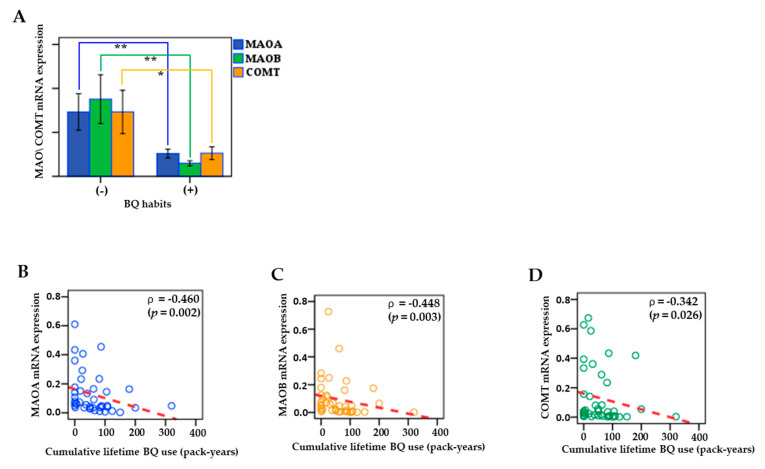
Associations between MAO/COMT mRNA and BQ use were analyzed in clinical samples. (**A**) MAO/COMT mRNA expression in patients with or without BQ habits. (**B**) The correlations of cumulative lifetime BQ use (pack-years) with the mRNA expression of MAOA. (**C**) The correlations of cumulative lifetime BQ use (pack-years) with the mRNA expression of MAOB. (**D**) The correlations of cumulative lifetime BQ use (pack-years) with the mRNA expression of COMT. Data are shown as mean ± SEM. Significant differences were analyzed with independent Mann–Whitney U test. *p < 0.05; **p < 0.01.

Figure 6 shows that the mRNA expressions of MAOA, MAOB, and COMT in patients with BQ chewing were significantly lower than those in patients without chewing (*p* < 0.05) (Figure 6A). Additionally, downregulation of MAO/COMT mRNA was linked to the increased amounts of cumulative BQ exposure in cancerous tissues. Low to moderate but significant negative correlations of cumulative lifetime in BQ users (pack-years) with the mRNA expression of MAOA (Spearman’s correlation, ρ = −0.460; *p* = 0.002), MAOB (Spearman’s correlation, ρ = −0.448; *p* = 0.003), and COMT (Spearman’s correlation, ρ = −0.342; *p* = 0.026) were observed in clinical samples (Figure 6B–D).

### 3.8. MAOA Expression in Human Oral Epithelial Cells

**Figure 7:**
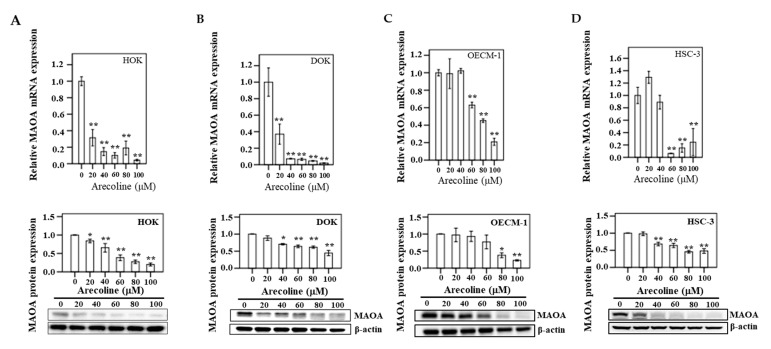
After 24 h of treatment of different concentrations of arecoline (0, 20, 40, 60, 80, and 100 µM), the mRNA and protein expression for MAOA in (**A**) HOK, (**B**) DOK, (**C**) OECM-1, and (**D**) HSC-3 are shown. Bars represent means ± standard error of the mean (* *p* < 0.05; ** *p* < 0.01). A representative result of Western blot analyses in three independent experiments is shown. * *p* < 0.05; ** *p* < 0.01.

The expression of mRNA and protein of MAOA was assayed in triplicate after HOK, DOK, OECM-1, and HSC-3 cells were treated with six different concentrations (0, 20, 40, 60, 80, and 100 µM) of arecoline for 24 h (Figure 7). In HOK, a significant decreasing trend was exhibited for MAOA mRNA, particularly at 100 µM arecoline (*p* < 0.05) compared to the control group (0 μM). Similarly, there was a significant change in the downregulation of MAOA at various concentrations of arecoline treatment in DOK cells (*p* < 0.05). In addition, in OECM-1 and HSC-3 cancer cells, compared to the control group, downregulation of MAOA mRNA and protein was found to be statistically significant after arecoline treatment (*p* < 0.05).

### 3.9. MAOB Expression in Human Oral Epithelial Cells

**Figure 8:**
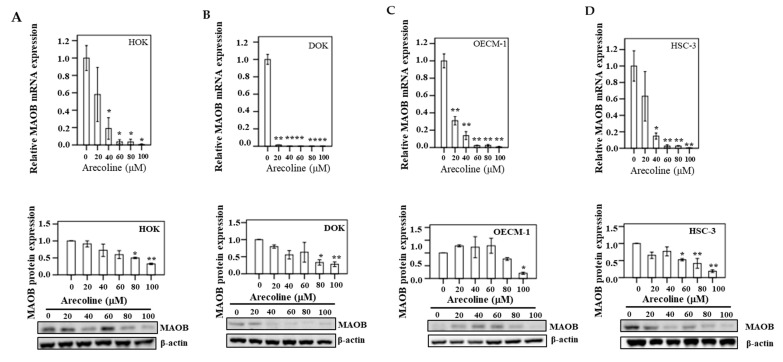
After 24 h of treatment of different concentrations of arecoline (0, 20, 40, 60, 80, and 100 µM), the mRNA and protein expression for MAOB in (**A**) HOK, (**B**) DOK, (**C**) OECM-1, and (**D**) HSC-3 are shown. Bars represent means ± standard error of the mean (* *p* < 0.05; ** *p* < 0.01). A representative result of Western blot analyses in three independent experiments is shown. * p < 0.05; ** *p* < 0.01.

After HOK, DOK, OECM-1, and HSC-3 cells were treated with different concentrations (0, 20, 40, 60, 80, and 100 µM) of arecoline for 24 h, the mRNA and protein expression of MAOB were assayed in triplicate (Figure 8). In HOK, a significant decreasing trend was exhibited for MAOB mRNA, particularly at 100 µM arecoline (*p* < 0.05) compared to the control group (0 μM). Similarly, there was a significant change in the downregulation of MAOB at various concentrations of arecoline treatment in DOK cells (*p* < 0.05). Additionally, in oral cancer cell lines (OECM-1 and HSC-3), compared to the control group, downregulation of MAOB mRNA and protein was found to be statistically significant after arecoline treatment (*p* < 0.05).

### 3.10. COMT Expression in Human Oral Epithelial Cells

**Figure 9:**
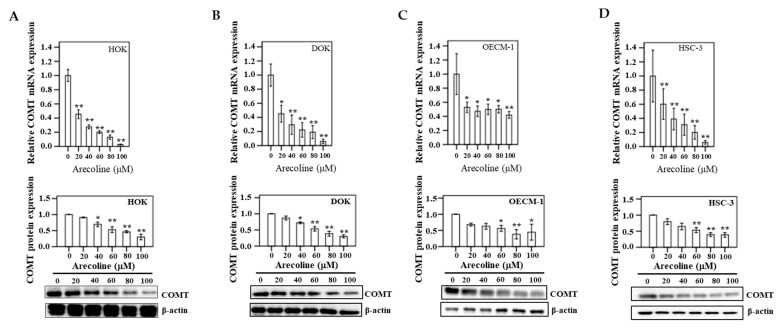
After 24 h treatment of different concentrations of arecoline (0, 20, 40, 60, 80, and 100 µM), the mRNA and protein for COMT in (**A**) HOK, (**B**) DOK, (**C**) OECM-1, and (**D**) HSC-3. Bars represent means ± standard error of the mean (*p < 0.05; **p < 0.01). A representative result of Western blot analyses in three independent experiments is shown.

COMT mRNA and protein expression were assayed in triplicate after DOK, OECM-1, and HSC-3 cells were treated with six different concentrations (0, 20, 40, 60, 80, and 100 µM) of arecoline for 24 h (Figure 9). In DOK, a significant decreasing trend was exhibited for COMT mRNA, particularly at 100 µM arecoline (*p* < 0.05), compared to the control group (0 μM). In addition, in oral cancer cell lines (OECM-1 and HSC-3), downregulation of COMT mRNA and protein was found to be statistically significant after arecoline treatment (*p* < 0.05) compared to the control group.

## 4. Discussion

The specific aim of this study was to examine the hypothesis that MAO and COMT variants are responsible for oral and pharyngeal cancers and OPMD risks in men. As mentioned above, there are several theoretical reasons for the potential implications of the MAO/COMT enzyme in OPMD and cancers of the oral cavity and pharynx, particularly among BQ chewers. BQ is not only an addictive and psychostimulant material but is also known as a carcinogen [26,27]. In 2004, the IARC monograph published comprehensive comments indicating that only chewing BQ, particularly without tobacco, was evaluated as a group 1 human carcinogen for the oral cavity, and its common component, AN, was also evaluated as group 1 human carcinogen and had sufficient evidence of carcinogenicity in experimental animals [27]. There are adequate findings with regard to BQ products and their association with elevated risk of OPMD and cancers of the oral cavity and pharynx [2,28]. Most evidence demonstrates that arecoline, the major alkaloid of AN, is the major cause of toxicity [27]. Indeed, previous IARC reports point out that arecoline has limited evidence for carcinogenicity [27]. Arecoline can inhibit the growth of different oral cells (e.g., human buccal epithelial cells, gingival fibroblasts, oral mucosal fibroblasts, and endothelial cells) and cause cell cycle arrest, apoptosis, and cytotoxicity [29,30,31,32,33].

A case–control study indicated that MAOA polymorphisms (rs144551722 SNP) were a significant biomarker of glioblastoma development in men [34]. There is a variety of reports on tumor suppression and the promotion of MAOA. The Gene Expression Omnibus (GEO) database has suggested a significant downregulation of MAOA in several cancerous tissues (such as lung cancer, kidney cancer, pulmonary adenocarcinoma, gastric cancer, and early hypopharyngeal cancer), compared with non-cancerous control tissues [13]. Moreover, previous studies have shown that the downregulation of MAOA may be associated with EBV-associated nasopharyngeal carcinoma [35], esophageal cancer [36], breast cancer [37], lymph node status (N0) of gastric cancer [38], cholangiocarcinoma [39,40], hepatocellular carcinoma [14], pheochromocytoma [41], and colon cancer [42]. These reports suggest that MAOA may function as a tumor suppressor through reducing biogenic amines that induce the progression of tumor through increased amine degradation [14]. In the analysis of MAOA polymorphism, a large case–control study indicated that the rs144551722 SNP of MAOA was a significant predictor of development of glioblastoma in men (*p* = 0.0056), but not in women, even after correction for multiple testing [34]. As mentioned above, our results consistently indicate a significant downregulation of MAOA in tumor tissues compared to adjacent non-tumor tissues. After arecoline treatment in the cell model (HOK, DOK, OECM-1, and HSC-3), both mRNA and protein levels of MAOA were significantly downregulated when compared to the control group. Furthermore, MAOA at-risk alleles (rs6323 [G], rs1137070 [T], or r5906957 [A]) were significantly responsible for the risk of oral and pharyngeal cancers and OPMD.

In contrast, MAOA was implied to have an oncogenic role by elevating intracellular oxidative stress. Higher expression of MAOA mediates hypoxia by increasing reactive oxygen species (ROS) in the tumorigenesis, progression, and metastasis of prostate cancer [43]. Prostate cancer patients with a high-grade tumor showed upregulation of MAOA expression [44,45]; the targeting of anti-depression drugs on MAOA may have potential for use in the therapy of advanced prostate cancer [46]. In addition, increased expression of MAOA has been identified in high-grade carcinomas of renal cell cancer [47]. In non-small-cell lung cancer (NSCLC), the expression of protein and mRNA levels of MAOA were higher in cancer tissues than those observed in adjacent non-cancerous tissues. Elevated MAOA expression in NSCLC tissues is associated with late-stage NSCLC and lymph node metastases [48]. In classical Hodgkin lymphoma, MAOA was expressed (181/241; 75%) by Hodgkin Reed–Sternberg (HRS) cells, with 34.8% showing strong expression [49].

While there is no explanation for the double-edged role of MAOA in different cancer types so far, we speculate that different cell context or cell type-specific gene expression in different types of cancer cells may have an influence on the tumor suppressor or oncoprotein activities of MAOA [50]. Further studies are required to settle this dispute.

In human hepatoma cells, suppression of MAOB activity significantly decreased endogenous levels of geranylgeranoic acid (GGA), an agent that prevents secondary primary hepatoma through oxidation of geranylgeraniol [51]. Likewise, our study demonstrated a significant downregulation of MAOB in tumor tissues when compared with their adjacent non-tumor tissues. In HOK, DOK, OECM-1, and HSC-3 cancer cells, compared to the control group, downregulation of MAOB mRNA and protein was found to be statistically significant after arecoline treatment. MAOB at-risk alleles (rs6324 (G)) were associated with the risk of oral and pharyngeal cancers and OPMD. To the best of our knowledge, the relationship between MAOB and cancer has rarely been mentioned. MAOB mRNA levels in human saliva were significantly downregulated in the oral cancer group compared to the non-tumor control group, suggesting its use as a potent biomarker for early detection of oral cancer [52]. In human endometrial carcinoma cells, MAOB is downregulated by high expression of miR-522 and accelerates the progression of endometrial carcinoma [53]. A previous study indicated a significant decrease in metabolic MAOB enzyme levels in head and neck squamous cell carcinoma (HNSCC) tissues compared to the control tissues (log2 fold change = −1.180; *p* = 4.59 E–12) [54]. Conversely, upregulation of MAOB was found in human gliomas [55] and colorectal cancer [56].

Previous reports have shown that polymorphisms in COMT are responsible for the risk of esophageal cancer [57]. COMT polymorphism is associated with the risk of lung cancer in non-smoking women [58]. Previous reports have shown that polymorphisms of COMT played a role in the risk of esophageal cancer [57,59], lung cancer [58], breast cancer [60,61,62], prostate cancer [63], and bladder cancer [59,64]. Although these reports focused on the respective cancers, there is little to no research on the relationship between COMT gene polymorphisms and oral and pharyngeal cancer risk, particularly in OPMD. The COMT SNP encodes a low activity that plays a potential role in the risk of breast cancer [61,65]. Methylation of the COMT gene can inactivate COMT and may result in the carcinogenesis of endometrial cancer [66]. However, some studies stated otherwise, where low enzyme activity of the COMT gene decreases the risk of bladder cancer among men [64]. Our study proved that the expression of the gene coding COMT was downregulated prominently in tumor tissues compared to adjacent non-tumor tissue, and the COMT at-risk genotypes (rs4633 (C/C) and rs9606186 (G/G)) were associated with risks of oral and pharyngeal cancers and OPMD, implying that COMT may play a key role in the development of oral and pharyngeal cancers and OPMD.

In our findings, the expression of COMT was significantly downregulated in oral and pharyngeal cancer tissues compared to non-cancerous tissue. In addition, we found a significant decreasing trend of COMT expression in the cell model with increasing concentrations of arecoline. Accordingly, the COMT enzyme may play a role as a tumor suppressor in breast and prostate cancers [67]. A previous report also indicated that the overexpression of COMT significantly decreased tumor invasion [68]. COMT overexpression can decrease cell proliferation and invasion in colorectal cancer [69]. Conversely, a significantly increased expression of COMT indicated that COMT could contribute to a putative risk in the formation of breast tumors [70]. In addition, overexpression of COMT has been found in pancreatic cancer tissue [71].

Although our results imply that MAO/COMT expression was significantly downregulated by increased BQ exposure, further studies are required to clarify the low to moderate negative correlation (−0.34 to −0.46) concern. MAOA/COMT were implied to have roles in the susceptibility of oral and pharyngeal cancers. Reactive oxygen species (ROS) generation has also been reported to mediate the cytotoxic effect of AN [72]. During the progression of oral and pharyngeal cancers, arecoline can induce the generation of ROS, resulting in DNA damage [73,74]. Additionally, arecoline can induce different phases of growth arrest in oral cancer cells via the ROS pathway [74]. ROS accumulation leads to oxidative DNA damage by producing DNA adducts, resulting in mutagenesis and malignant cell transformation to oral and pharyngeal cancers [75]. Notably, during mitochondrial oxidative metabolism, cell necrosis is induced by a surge in reactive oxygen species (ROS), which may be yielded through MAO catalysis [7]. Interestingly, COMT also plays a key role in inhibiting ROS formation through methylation of catechol estrogens [76]. Our findings highlight the association of MAO and COMT biomarkers in inducing the risks of OPMD and cancers of the oral cavity and pharynx. Further studies are needed to dissect the potential ROS pathway between MAO and COMT and the possible molecular mechanisms implicated in the development of cancers of the oral cavity and pharynx.

### Study Limitation

One limitation of this study was the lack of human papillomavirus (HPV) data in this study population. HPV has been associated with development of oropharyngeal squamous cell carcinoma and is different from BQ-related oropharyngeal squamous cell carcinoma [77]. In this study, male patients had 86.5% prevalence of BQ chewing, while the prevalence of HPV in Taiwanese male BQ chewers was 3% [78]. Therefore, we did not include HPV issue in our study. However, there are also reports showing the increasing prevalence of HPV in Taiwan [77,78] and worthy of further extensive studies to explore its role in the pathogenesis of oral cavity and pharynx cancers. Additionally, BQ chewing mainly has deleterious effects on the oral cavity [79]. Therefore, we only focused our in vitro studies using normal (HOK), pre-cancerous (DOK), and cancerous (OECM1 and HSC-3) from the oral cavity.

## 5. Conclusions

Our study is the first to demonstrate the associations between MAO and COMT in BQ-related OPMD and cancers of the oral cavity and pharynx. Our findings support the hypothesis that genetic variations and downregulation of MAO and COMT may play a putative role in the development of OPMD and cancers of the oral cavity and pharynx in men.

## Figures and Tables

**Table 1:** Distribution of selected demographic characteristics and substance use among males with oral and pharyngeal cancers, OPMD, and control (*n* = 530).

	Oral and Pharyngeal Cancers (*n* = 297)		OPMD (*n* = 40)		Control (*n* = 193)		*p*-Value
	Mean ± SD		Mean ± SD		Mean ± SD		
Demography and Substance use Factors	*n*	(%) ^a^	*n*	(%)	*n*	(%)	
**Age, years (mean ± SD)**	53.97 ± 10.32 ^†^		50.83 ± 11.57		46.08 ± 12.94		<0.001 *^b^
Age group ≤ 50 (years)	117	(39.4)	22	(55.0)	127	(65.8)	<0.001 *^c^
Age group > 50 (years)	180	(60.6)	18	(45.0)	66	(34.2)	
**BMI (kg/m^2^)**	24.58 ± 4.07		26.52 ± 4.14		26.27 ± 27.74		0.668 ^b^
**Ethnicity**							
Minnan	220	(74.1)	25	(62.5)	158	(81.9)	0.016 *^c^
Non-Minnan	77	(25.9)	15	(37.5)	35	(18.1)	
**Marital status**							
Unmarried	41	(13.8)	7	(17.5)	13	(6.7)	0.027 *^c^
Married	256	(86.2)	33	(82.5)	180	(93.3)	
**Education level**							
Low (≤9 years)	197	(66.3)	25	(62.5)	84	(43.5)	<0.001 *^c^
High (>9 years)	100	(33.7)	15	(37.5)	109	(56.5)	
**Alcohol drinking status**							
*Never*	71	(23.9)	13	(32.5)	96	(49.7)	<0.001 *^c^
*Current or former*	226	(76.1)	27	(67.5)	97	(50.3)	
Age at starting drinking (years)	22.61 ± 6.61		20.25 ± 3.95		20.68 ± 7.72		0.047 *^b^
Years of drinking	27.91 ± 10.81^†^		27.22 ± 12.91		22.12 ± 11.35		<0.001 ^b^
*Former*							
Age at quitting drinking (years)	48.58 ± 11.55 ^†^		39.00 ± 12.61		38.47 ± 11.73		<0.001 ^b^
Years of quitting alcohol	8.86 ± 7.90		12.57 ± 14.49		7.44 ± 6.97		0.249 ^b^
**BQ chewing status**							
*Never*	40	(13.5)	7	(17.5)	130	(67.4)	<0.001 *^c^
*Current or former*	257	(86.5)	33	(82.5)	63	(32.6)	
**Age at starting chewing (years)**	22.44 ± 6.39^†^		22.66 ± 8.26^‡^		19.00 ± 4.86		<0.001 *^b^
**Years of chewing**	24.61 ± 10.48^†^		21.72 ± 9.64		19.97 ± 10.74		0.007 *^b^
Average amount of chewing (quid)	28.96 ± 27.12		40.43 ± 36.91^‡^		20.18 ± 14.31		0.006 *^b^
Cumulative lifetime BQ use (pack-years)	76.10 ± 89.07^†^		86.24 ± 83.87		41.56 ± 37.10		0.016 *^b^
**Type of BQ material**							
BQ with inflorescence of Piper betel Linn.	27	(10.5)	3	(9.1)	13	(20.6)	0.093^c^
BQ with Piper betel leaf	186	(72.4)	23	(69.7)	35	(55.6)	
Mixed	44	(17.1)	7	(21.2)	15	(23.8)	
**BQ juice swallowing**							
Swallowed	109	(42.4)	15	(45.5)	9	(14.3)	<0.001 *^c^
Never swallowed	148	(57.6)	18	(54.6)	54	(85.7)	
*Former*							
**Age at quitting chewing (years)**	45.45 ± 11.49 ^†^		42.00 ± 8.82		36.84 ± 9.76		<0.001 *^b^
**Years of quitting BQ**	10.15 ± 7.55		11.33 ± 9.31		9.60 ± 8.76		0.792 ^b^
**Cigarette smoking status**							
*Never*	36	(12.1)	5	(12.5)	43	(22.3)	0.009 *^c^
*Current or former*	261	(87.9)	35	(87.5)	150	(77.7)	
Age at starting smoking (years)	19.53 ± 4.02 ^†^		18.31 ± 3.81		18.29 ± 5.15		0.026 *^b^
Years of smoking	29.81 ± 11.42 ^†^		28.16 ± 11.56		24.21 ± 10.57		<0.001 *^b^
Average amount of smoking	23.88 ± 13.73 ^†^		29.41 ± 16.74 ^‡^		15.51 ± 10.67		<0.001 *^b^
Cumulative lifetime cigarette use (pack-years)	37.04 ± 25.83		41.52 ± 27.06		27.33 ± 19.49		0.066 ^b^
*Former*							
**Age at quitting smoking (years)**	46.96 ± 11.87 ^†^		43.33 ± 13.50		38.74 ± 14.34		0.009 *^b^
**Years of quitting cigarette**	10.42 ± 9.69		17.83 ± 13.42		14.78 ± 13.03		0.076 ^b^

Abbreviations: OPMD, oral potentially malignant disorder; BQ, betel quid. ^a^ May not total 100% due to rounding. ^b^ Significant difference was tested by the general linear (GLM) model (* *p* < 0.05). ^c^ Significant difference was tested by Chi-square analysis (* *p* < 0.05). OPMD: oral potential malignant disorders; SD: standard deviation; BMI: body mass index; BQ: betel quid. Cumulative BQ exposure (pack-years) was defined as the daily number of packs (number of betel quid/10) consumed multiplied by the years of BQ chewing habits. One pack was denoted as 10 quids. Cumulative cigarette exposure (pack-years) was defined as the daily number of packs (number of cigarette/20) consumed multiplied by the years of cigarette smoking habits. One pack was denoted as 20 cigarettes. ^†^ Compares oral and pharyngeal cancer cases versus controls by the post hoc comparison of Bonferroni pairs comparison (*p* < 0.05). ^‡^ Compares OPMD cases versus controls by the post hoc comparison of Bonferroni pairs comparison (*p* < 0.05).

**Table 2:** The synergistic effects of BQ chewing and susceptibility SNPs of MAO/COMT on oral and pharyngeal cancers and OPMD were calculated by stratifying the uses of BQ across the susceptibility SNPs of MAO/COMT (*n* = 530).

		Oral and Pharyngeal Cancers (*n* = 297)		OPMD (*n* = 40)		Control (*n* = 193)		Oral and Pharyngeal Cancers vs. Control			OPMD vs. Control		
								Risk Assessment			Risk Assessment		
SNPs	BQ	*n*	(%) ^a^	*n*	(%)	*n*	(%)	AOR	(95% CI) ^b^	*p*	AOR	(95% CI) ^b^	*p*
***MAOA***													
rs6323													
T		98	(33.0)	10	(25.0)	89	(46.1)	1.00	(Reference)		1.00	(Reference)	
G		199	(67.0)	30	(75.0)	104	(53.9)	1.98	(1.23–3.20) *	0.005	2.89	(1.27–6.58) *	0.012
T	(−)	14	(4.7)	5	(12.5)	57	(29.5)	1.00			1.00	(Reference)	
G	(−)	26	(8.8)	2	(5.0)	73	(37.8)	1.72	(0.77–3.87)	0.187	0.37	(0.07–2.01)	0.247
T	(+)	84	(28.3)	5	(12.5)	32	(16.6)	12.67	(5.44–29.53) *	<0.001	1.95	(0.45–8.54)	0.374
G	(+)	173	(58.3)	28	(70.0)	31	(16.1)	31.15	(13.43–72.27) *	<0.001	12.77	(3.61–45.20) *	<0.001
rs1137070													
C		97	(32.7)	10	(25.0)	103	(53.4)	1.00	(Reference)		1.00	(Reference)	
T		200	(67.3)	30	(75.0)	90	(46.6)	2.37	(1.47–3.81) *	<0.001	3.25	(1.43–7.38) *	0.005
C	(−)	14	(4.7)	5	(12.5)	70	(36.3)	1.00	(Reference)		1.00	(Reference)	
T	(−)	26	(8.8)	2	(5.0)	60	(31.1)	2.30	(1.03–5.13) *	0.043	0.52	(0.10–2.86)	0.455
C	(+)	83	(28.0)	5	(12.5)	33	(17.1)	13.21	(5.90–29.57) *	<0.001	2.32	(0.56–9.67)	0.249
T	(+)	174	(58.6)	28	(70.0)	30	(15.5)	36.22	(16.24–80.78) *	<0.001	15.47	(4.67–51.26) *	<0.001
rs5906957													
G		104	(35.0)	10	(25.0)	79	(40.9)	1.00	(Reference)		1.00	(Reference)	
A		193	(65.0)	30	(75.0)	114	(59.1)	1.67	(1.03–2.71) *	0.036	2.55	(1.11–5.85) *	0.027
G	(−)	15	(5.1)	5	(12.5)	48	(24.9)	1.00	(Reference)		1.00	(Reference)	
A	(−)	25	(8.4)	2	(5.0)	82	(42.5)	1.24	(0.55–2.80)	0.598	0.27	(0.05–1.50)	0.136
G	(+)	89	(30.0)	5	(12.5)	31	(16.1)	10.70	(4.57–25.03) *	<0.001	1.58	(0.36–7.01)	0.547
A	(+)	168	(56.6)	28	(70.0)	32	(16.6)	24.15	(10.44–55.89) *	<0.001	10.04	(2.83–35.56) *	<0.001
***MAOB***													
rs6324													
A		74	(24.9)	4	(10.0)	157	(81.4)	1.00	(Reference)		1.00	(Reference)	
G		223	(75.1)	36	(90.0)	36	(18.7)	13.00	(7.35–22.98) *	<0.001	37.45	(11.94–117.46) *	<0.001
A	(−)	5	(1.7)	1	(2.5)	108	(56.0)	1.00	(Reference)		1.00	(Reference)	
G	(−)	35	(11.8)	6	(15.0)	22	(11.4)	30.24	(10.19–89.73) *	<0.001	29.73	(3.33–265.50) *	0.002
A	(+)	69	(23.2)	3	(7.5)	49	(25.4)	35.34	(12.14–102.86) *	<0.001	8.12	(0.76–86.96)	0.084
G	(+)	188	(63.3)	30	(75.0)	14	(7.3)	307.03	(97.43–967.54) *	<0.001	246.94	(28.08–NA) *	<0.001
rs1799836													
C		50	(16.8)	5	(12.5)	34	(17.6)	1.00	(Reference)		1.00	(Reference)	
T		247	(83.2)	35	(87.5)	159	(82.4)	1.36	(0.75–2.47)	0.309	1.97	(0.68–5.73)	0.213
C	(−)	5	(1.7)	2	(5.0)	19	(9.8)	1.00	(Reference)		1.00		
T	(−)	35	(11.8)	5	(12.5)	111	(57.5)	1.46	(0.47–4.59)	0.513	0.44	(0.08–2.53)	0.354
C	(+)	45	(15.2)	3	(7.5)	15	(7.8)	15.67	(4.40–55.86) *	<0.001	1.93	(0.25–14.87)	0.530
T	(+)	212	(71.4)	30	(75.0)	48	(24.9)	22.60	(7.13–71.62) *	<0.001	6.32	(1.21–33.08) *	0.029
rs3027452													
A		41	(13.8)	5	(12.5)	27	(14.0)	1.00	(Reference)		1.00	(Reference)	
G		256	(86.2)	35	(87.5)	166	(86.0)	1.38	(0.73–2.62)	0.329	1.57	(0.53–4.64)	0.417
A	(−)	4	(1.4)	2	(5.0)	13	(6.7)	1.00	(Reference)		1.00	(Reference)	
G	(−)	36	(12.1)	5	(12.5)	117	(60.6)	1.14	(0.32–4.13)	0.841	0.26	(0.04–1.59)	0.145
A	(+)	37	(12.5)	3	(7.5)	14	(7.3)	11.51	(2.78–47.73) *	<0.001	1.38	(0.17–11.12)	0.765
G	(+)	220	(74.1)	30	(75.0)	49	(25.4)	18.57	(5.06–68.18) *	<0.001	4.00	(0.73–22.03)	0.112
***COMT***													
rs4633													
T/T		15	(5.1)	4	(10.0)	17	(8.8)	1.00	(Reference)		1.00	(Reference)	
C/T		91	(30.6)	7	(17.5)	81	(42.0)	1.05	(0.40–2.76)	0.920	0.30	(0.07–1.26)	0.100
C/C		191	(64.3)	29	(72.5)	95	(49.2)	1.86	(0.74–4.71)	0.189	1.06	(0.29–3.81)	0.931
T/T+C/T		106	(35.7)	11	(27.5)	98	(50.8)	1.00	(Reference)		1.00	(Reference)	
C/C		191	(64.3)	29	(72.5)	95	(49.2)	1.78	(1.11–2.85) *	0.016	2.65	(1.19–5.88) *	0.017
T/T+C/T	(−)	13	(4.4)	2	(5.0)	68	(35.2)	1.00	(Reference)		1.00	(Reference)	
C/C	(−)	27	(9.1)	5	(12.5)	62	(32.1)	1.82	(0.81–4.07)	0.145	2.55	(0.47–13.92)	0.280
T/T+C/T	(+)	93	(31.3)	9	(22.5)	30	(15.5)	15.38	(6.81–34.72) *	<0.001	10.12	(1.84–55.53) *	0.008
C/C	(+)	164	(55.2)	24	(60.0)	33	(17.1)	27.14	(12.16–60.56) *	<0.001	26.86	(5.24–137.65) *	<0.001
rs9605030													
C/C		177	(59.6)	16	(40.0)	115	(59.6)	1.00	(Reference)		1.00	(Reference)	
C/T		103	(34.7)	20	(50.0)	69	(35.8)	1.06	(0.65–1.73)	0.829	2.38	(1.09–5.21) *	0.030
T/T		17	(5.7)	4	(10.0)	9	(4.7)	1.07	(0.38–3.00)	0.904	3.29	(0.81–13.36)	0.096
C/C+C/T		280	(94.3)	36	(90.0)	184	(95.3)	1.00	(Reference)		1.00	(Reference)	
T/T		17	(5.7)	4	(10.0)	9	(4.7)	1.04	(0.38–2.90)	0.935	2.23	(0.58–8.51)	0.242
C/C+C/T	(−)	40	(13.5)	5	(12.5)	123	(63.7)	1.00	(Reference)		1.00	(Reference)	
T/T	(−)	0	(0.0)	2	(5.0)	7	(3.6)	NA		0.983	4.18	(0.61–28.75)	0.147
C/C+C/T	(+)	240	(80.8)	31	(77.5)	61	(31.6)	13.77	(7.72–24.54) *	<0.001	12.58	(3.98–39.83) *	<0.001
T/T	(+)	17	(5.7)	2	(5.0)	2	(1.0)	31.57	(6.10–163.34) *	<0.001	32.29	(3.28–317.97) *	0.003
rs9606186													
C/C		22	(7.4)	3	(7.5)	23	(11.9)	1.00	(Reference)		1.00	(Reference)	
G/C		125	(42.1)	12	(30.0)	84	(43.5)	2.28	(1.03–5.02) *	0.042	1.60	(0.38–6.65)	0.521
G/G		150	(50.5)	25	(62.5)	86	(44.6)	2.72	(1.25–5.94) *	0.012	3.29	(0.84–12.88)	0.087
C/C+G/C		147	(49.5)	15	(37.5)	107	(55.4)	1.00	(Reference)		1.00	(Reference)	
G/G		150	(50.5)	25	(62.5)	86	(44.6)	1.41	(0.89–2.24)	0.144	2.29	(1.08–4.84) *	0.031
C/C+G/C	(−)	19	(6.4)	3	(7.5)	70	(36.3)	1.00	(Reference)		1.00	(Reference)	
G/G	(−)	21	(7.1)	4	(10.0)	60	(31.1)	1.44	(0.66–3.13)	0.364	1.53	(0.32–7.27)	0.594
C/C+G/C	(+)	128	(43.1)	12	(30.0)	37	(19.2)	15.38	(7.44–31.82) *	<0.001	8.13	(1.93–34.27) *	0.004
G/G	(+)	129	(43.4)	21	(52.5)	26	(13.5)	22.13	(10.23–47.84) *	<0.001	20.73	(4.97–86.52) *	<0.001

Abbreviations: AOR, adjusted odds ratio; BQ, betel quid; OPMD, oral potentially malignant disorder; SNP, single nucleotide polymorphism; NA, non-applicable owing to limited samples. ^a^ May not total 100% due to rounding. ^b^ AOR was obtained after adjustment for age, ethnicity, marital status, educational level, and covariates (alcohol, betel quid, and cigarette uses). * *p* < 0.05.

**Table 3:** Joint effects of susceptibility SNPs of MAO/COMT for the occurrence risks of oral and pharyngeal cancers and OPMD (*n* = 530).

			Oral and Pharyngeal Cancers (*n* = 297)		OPMD (*n* = 40)		Control (*n* = 193)		Oral and Pharyngeal Cancers vs. Control			OPMD vs. Control		
									Risk Assessment			Risk Assessment		
SNPs			*n*	(%) ^a^	*n*	(%)	*n*	(%)	AOR	(95% CI) ^b^	*p*	AOR	(95% CI) ^b^	*p*
***MAOA***	***MAOB***													
rs6323	rs6324													
T	A		19	(6.4)	2	(5.0)	74	(38.3)	1.00	(Reference)		1.00	(Reference)	
G	A		55	(18.5)	2	(5.0)	83	(43.0)	3.16	(1.49–6.71) *	0.003	1.05	(0.14–7.98)	0.962
T	G		79	(26.6)	8	(20.0)	15	(7.8)	22.53	(8.96–56.70) *	<0.001	20.18	(3.63–112.27) *	0.001
G	G		144	(48.5)	28	(70.0)	21	(10.9)	30.32	(13.07–70.35) *	<0.001	53.21	(10.91–259.62) *	<0.001
***MAOA***	***MAOB***													
rs1137070	rs6324													
C	A		19	(6.4)	2	(5.0)	81	(42.0)	1.00	(Reference)		1.00	(Reference)	
T	A		55	(18.5)	2	(5.0)	76	(39.4)	3.28	(1.53–7.01) *	0.002	0.90	(0.12–6.84)	0.919
C	G		78	(26.3)	8	(20.0)	22	(11.4)	15.53	(6.55–36.83) *	<0.001	12.37	(2.31–66.20) *	0.003
T	G		145	(48.8)	28	(70.0)	14	(7.3)	45.56	(18.51–112.17) *	<0.001	70.59	(14.17–351.78) *	<0.001
***MAOA***	***COMT***													
rs6323	rs4633													
T	T/T+C/T		44	(14.8)	5	(12.5)	42	(21.8)	1.00	(Reference)		1.00	(Reference)	
T	C/C		54	(18.2)	5	(12.5)	47	(24.4)	1.18	(0.57–2.46)	0.659	1.09	(0.27–4.31)	0.906
G	T/T+C/T		62	(20.9)	6	(15.0)	56	(29.0)	1.34	(0.65–2.77)	0.422	1.25	(0.33–4.69)	0.745
G	C/C		137	(46.1)	24	(60.0)	48	(24.9)	2.94	(1.50–5.76) *	0.002	4.74	(1.54–14.57) *	0.007
***MAOA***	***COMT***													
rs1137070	rs4633													
C	T/T+C/T		44	(14.8)	5	(12.5)	54	(28.0)	1.00	(Reference)		1.00	(Reference)	
C	C/C		53	(17.9)	5	(12.5)	49	(25.4)	1.47	(0.72–2.99)	0.293	1.26	(0.32–4.90)	0.744
T	T/T+C/T		62	(20.9)	6	(15.0)	44	(22.8)	2.02	(0.99–4.14)	0.053	1.63	(0.44–6.10)	0.465
T	C/C		138	(46.5)	24	(60.0)	46	(23.8)	3.57	(1.86–6.82) *	<0.001	5.31	(1.76–16.00) *	0.003
***MAOB***	***COMT***													
rs6324	rs4633													
A	T/T+C/T		18	(6.1)	0	(0.0)	77	(39.9)	1.00	(Reference)		1.00	(Reference)	
A	C/C		56	(18.9)	4	(10.0)	80	(41.5)	2.63	(1.22–5.67) *	0.013	NA		
G	T/T+C/T		88	(29.6)	11	(27.5)	21	(10.9)	15.19	(6.30–36.60) *	<0.001	NA		
G	C/C		135	(45.5)	25	(62.5)	15	(7.8)	39.27	(15.66–98.47) *	<0.001	NA		
***MAOA***	***MAOB***	***COMT***												
rs6323	rs6324	rs4633												
T	A	T/T+C/T	8	(2.7)	0	(0.0)	34	(17.6)	1.00	(Reference)		1.00	(Reference)	
T	A	C/C	11	(3.7)	2	(5.0)	40	(20.7)	1.23	(0.35–4.27)	0.746	NA		
G	A	T/T+C/T	10	(3.4)	0	(0.0)	43	(22.3)	1.43	(0.39–5.18)	0.591	NA		
G	A	C/C	45	(15.2)	2	(5.0)	40	(20.7)	5.24	(1.72–15.98) *	0.004	NA		
T	G	T/T+C/T	36	(12.1)	5	(12.5)	8	(4.2)	17.76	(4.69–67.25) *	<0.001	NA		
G	G	T/T+C/T	52	(17.5)	6	(15.0)	13	(6.7)	19.31	(5.53–67.46) *	<0.001	NA		
T	G	C/C	43	(14.5)	3	(7.5)	7	(3.6)	36.43	(9.08–146.12) *	<0.001	NA		
G	G	C/C	92	(31.0)	22	(55.0)	8	(4.2)	56.99	(15.82–205.31) *	<0.001	NA		
***MAOA***	***MAOB***	***COMT***												
rs1137070	rs6324	rs4633												
C	A	T/T+C/T	8	(2.7)	0	(0.0)	41	(21.2)	1.00	(Reference)		1.00	(Reference)	
C	A	C/C	11	(3.7)	2	(5.0)	40	(20.7)	1.44	(0.41–5.03)	0.570	NA		
T	A	T/T+C/T	10	(3.4)	0	(0.0)	36	(18.7)	1.74	(0.48–6.33)	0.404	NA		
T	A	C/C	45	(15.2)	2	(5.0)	40	(20.7)	5.68	(1.85–17.42) *	0.002	NA		
C	G	T/T+C/T	36	(12.1)	5	(12.5)	13	(6.7)	11.21	(3.23–38.89) *	0.001	NA		
C	G	C/C	42	(14.1)	3	(7.5)	9	(4.7)	35.29	(9.09–136.95) *	<0.001	NA		
T	G	T/T+C/T	52	(17.5)	6	(15.0)	8	(4.2)	40.69	(10.47–158.05) *	<0.001	NA		
T	G	C/C	93	(31.3)	22	(55.0)	6	(3.1)	78.62	(20.37–303.45) *	<0.001	NA		

Abbreviations: AOR, adjusted odds ratio; BQ, betel quid; OPMD, oral potentially malignant disorder; SNP, single nucleotide polymorphism; NA, not applicable owing to limited samples. ^a^ May not total 100% due to rounding. ^b^ AOR was obtained after adjustment for age, ethnicity, marital status, educational level, and covariates (alcohol, betel quid, and cigarette uses). NA, non-applicable owing to limited samples (*n* = 0). * *p* < 0.05.

## Data Availability

The data presented in this study are available on request from the corresponding author.

## Supplementary Materials

The following are available online at https://www.mdpi.com/article/10.3390/cancers13133268/s1, Figure S1: HOK cell viability after arecoline treatment for 24 h was evaluated by MTT assay in triplicates (mean ± SEM). The asterisks presented the statistically significant difference (*p* < 0.05) comparing to control (0 μM). Figure S2: Scatter plots showing the correlation between MAOA, MAOB, and COMT mRNA expression in the same cancer tissues (*n* = 42). Table S1. Sequence and labeled dye information of primer and probes for genotyping assay. Table S2. The primers of MAO/COMT used in qRT-PCR. Table S3. The synergistic effects of BQ chewing and higher susceptibility SNPs of MAO/COMT on oral cancer, pharyngeal cancers and OPMD were calculated by stratifying the uses of BQ across the susceptibility SNPs of MAO/COMT (*n* = 530). Table S4. The synergistic effects of cigarette smoking and susceptibility SNPs of MAO/COMT on oral and pharyngeal cancers and OPMD were calculated by stratifying the uses of cigarette across the susceptibility SNPs of MAO/COMT (*n* = 530). Table S5. The synergistic effects of alcohol drinking and susceptibility SNPs of MAO/COMT on oral and pharyngeal cancers and OPMD were calculated by stratifying the uses of alcohol across the susceptibility SNPs of MAO/COMT (*n* = 530). Table S6. Distribution of significantly demographic characteristics and substance use among males with oral and pharyngeal cancers and control were calculated after propensity-score matching for age (*n* = 300). Table S7. The susceptibility SNPs of MAO/COMT on oral and pharyngeal cancers were calculated after propensity-score matching for age (*n* = 300). Table S8. Distribution of selected clinical characteristics of males with oral and pharyngeal cancers between oral and pharynx sites (*n* = 42). Table S9. Associations between MAOA, MAOB, and COMT mRNA expression (*n* = 42).
