# Supplementary material for: Genetic and Proteinic Linkage of MAO and COMT with Oral Potentially Malignant Disorders and Cancers of the Oral Cavity and Pharynx
Source: Cancers (Basel). 2021 Jun 29;13(13):3268. doi: 10.3390/cancers13133268 (PMC8268107; doi:10.3390/cancers13133268)
Supplement: Supplementary file 1 [file cancers-13-03268-s001.zip › cancers-1218087-supplementary.pdf]

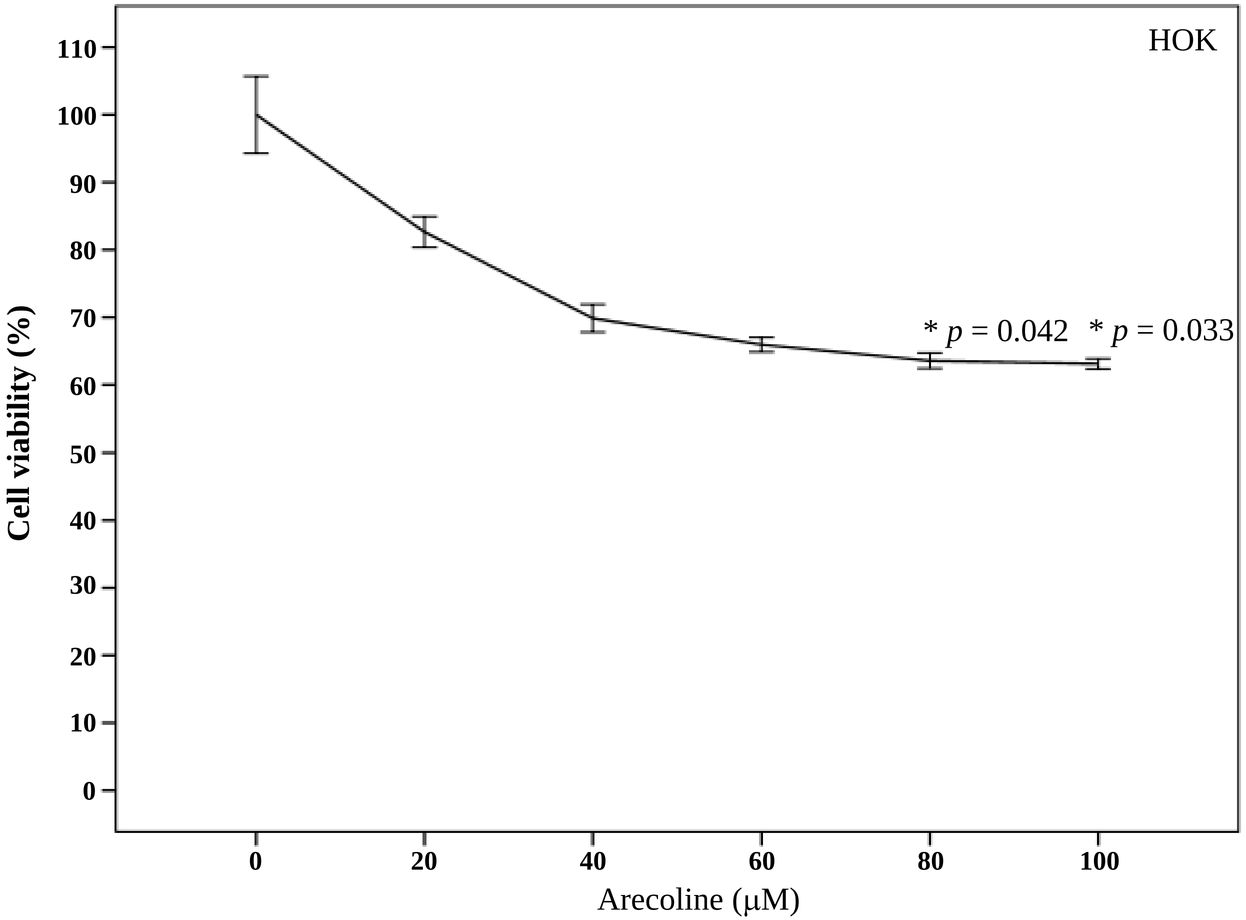

Figure S1. HOK cell viability after arecoline treatment for 24 h was evaluated by MTT assay in triplicates (mean ± SEM). The asterisks presented the statistically significant difference ($p < 0.05$) comparing to control (0 μM).

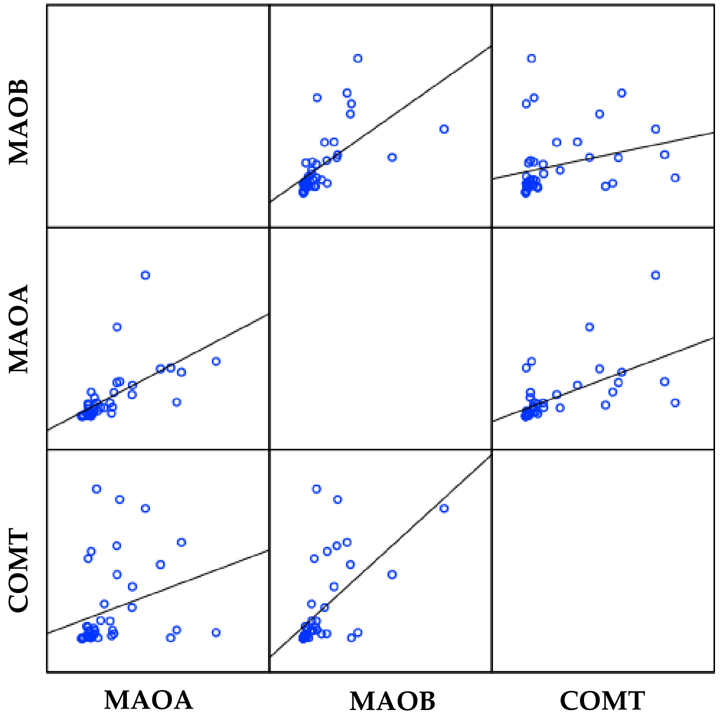

Figure S2. Scatter plots showing the correlation between MAOA, MAOB, and COMT mRNA expression in the same cancer tissues (n = 42)

Supplementary Materials:

Table S1. Sequence and labeled dye information of primer and probes for genotyping assay

SNP	Gene	Name	5' labeled	Sequence	3' labeled	QPCR Working Conc.
**rs6323**	MAOA	rs6323-F	----	TCTGAACCTAGGATGTCCCTGC	----	500 nM
		rs6323-R	----	GTCAATCCAATCAGCCTGAGC	----	500 nM
		rs6323-VIC-P	VIC	ACGGAGGGCAAATGT	BHQ1	200 nM
		rs6323-FAM-P	FAM	CACGGAAGGCAAATGT	BHQ1	200 nM
**rs1137070**	MAOA	rs1137070-F	----	TTTCAGGTCTTAAATGGTCTCGG	----	500 nM
		rs1137070-R	----	GCCCAGAGTCACCAAACTTACC	----	500 nM
		rs1137070-VIC-P	VIC	CCAGATATCTTTC	MGB	200 nM
		rs1137070-FAM-P	FAM	CAGATGTCTTTCT	MGB	200 nM
**rs5906957**	MAOA	rs5906957-F	----	TAGTGCATCAGTGAGAGTGAGCCT	----	500 nM
		rs5906957-R	----	CTTGGCTGTCAGTGAGAAGGG	----	500 nM
		rs5906957-VIC-P	VIC	AGGTCTCGAGCAG	MGB	200 nM
		rs5906957-FAM-P	FAM	AGGTCTTGAGCAG	MGB	200 nM
**rs6324**	MAOB	rs6324-F	----	TCTGAACCTAGGATGTCCCTGC	----	500 nM
		rs6324-R	----	GTCAATCCAATCAGCCTGAGC	----	500 nM
		rs6324-VIC-P	VIC	ACGGAGGGCAAATGTC	BHQ1	200 nM
		rs6324-FAM-P	FAM	CACGGAAGGCAAATGTC	BHQ1	200 nM
**rs1799836**	MAOB	rs1799836-F	----	CAGTTACTTAGTCCTTTAGGGAGCAG	----	500 nM
		rs1799836-R	----	GCATGCAGGATCTGAAATGAAAG	----	500 nM
		rs1799836-VIC-P	VIC	AGATGGTGTCACTTTTGC	BHQ1	200 nM
		rs1799836-FAM-P	FAM	AGATGGTGTCGCTTTTG	BHQ1	200 nM
**rs3027452**	MAOB	rs3027452-F	----	CTTTTGCTTGACATTGGCAGG	----	500 nM
		rs3027452-R	----	GATAGTTTGCTTTCTGGTGTGCTTC	----	500 nM
		rs3027452-VIC-P	VIC	TGGTGAGAGGTTATCACC	BHQ1	200 nM
		rs3027452-FAM-P	FAM	ATGGTGAGAGATTATCACCT	BHQ1	200 nM
**rs4633**	COMT	rs4633-F	----	ACCTGCTCATGGGTGACACC	----	500 nM
		rs4633-R	----	TGTCAATGGCCTCCAGCAC	----	500 nM
		rs4633-VIC-P	VIC	AGCACGTGGTTCA	MGB	200 nM
		rs4633-FAM-P	FAM	AGCACATGGTTCA	MGB	200 nM
**rs9605030**	COMT	rs9605030-F	----	GCAGCTGTCTGTATGGATGTGC	----	500 nM
		rs9605030-R	----	TATGCTTGCAAACGACTGCC	----	500 nM
		rs9605030-VIC-P	VIC	ATCAGCCCGAGAG	MGB	200 nM
		rs9605030-FAM-P	FAM	ATCAGCCCAAGAG	MGB	200 nM
**rs9606186**	COMT	rs9606186-F	----	AGGGTCTGTCATACAAGGCTGG	----	500 nM
		rs9606186-R	----	AGATAAGTGCAGCCTTCTCATGG	----	500 nM
		rs9606186-VIC-P	VIC	ACACCTGCATCAG	MGB	200 nM
		rs9606186-FAM-P	FAM	AACACCTCCATCAG	MGB	200 nM

Table S2. The primers of MAO/COMT used in qRT-PCR

Primer ID	organism	gene name	gene symbol	genbank accession/ Tiger bank ID for pig	Sequence Length	Product Length	Sense Primer	Position	Length	Tm	Anti-sense Primer	Position	Length	Tm
HT00633	Human	Homo sapiens monoamine oxidase A (MAOA)	MAOA	NM_000240	5,330	78	CCTCATCCTCCACTGAAGAGTATG	5119	24	64.9	ACATTAGGCACCAAGTTCACAATC	5196	24	64.9
HT00634	Human	Homo sapiens monoamine oxidase B (MAOB), mRNA.	MAOB	NM_000898	2,611	112	TTTGTGCGGTGTGTCTCCAG	2454	20	59.7	CCAAGTGTGTACTGTCCTTTGTATG	2565	25	59.5
HT00641	Human	Homo sapiens catechol-O-methyltransferase (COMT)	COMT	NM_000754	2272	102	CCCTGCTGCCCTTGACTTG	1360	19	68.2	GCGTGCCACCCAGAACC	1461	17	68.2

Table S3. The synergistic effects of BQ chewing and higher susceptibility SNPs of MAO/COMT on oral cancer, pharyngeal cancers and OPMD were calculated by stratifying the uses of BQ across the susceptibility SNPs of MAO/COMT (N = 530)

		**Oral cancer (N = 209)**	**Pharyngeal cancer (N = 88)**	**OPMD (N = 40)**	**Control (N = 193)**	**Oral cancer vs. control**		**Pharyngeal cancer vs. control**		**OPMD vs. control**	
						Risk assessment		Risk assessment		Risk assessment	
SNPs	BQ	N (%)	N (%)	N (%)	N (%)	AOR (95%CI)[a]	*p*	AOR (95% CI)[a]	*p*	AOR (95% CI)[a]	*p*
MAOA											
rs6323											
T		72 (34.5)	26 (29.6)	10 (25.0)	89 (46.1)	1.00 [Reference]		1.00 [Reference]		1.00 [Reference]	
G		137 (65.6)	62 (70.5)	30 (75.0)	104 (53.9)	1.80 (1.09-2.96)*	0.022	2.75 (1.42-5.32)*	0.003	2.97 (1.30-6.79)*	0.010
T	(-)	9 (4.3)	5 (5.7)	5 (12.5)	57 (29.5)	1.00 [Reference]		1.00 [Reference]		1.00 [Reference]	
G	(-)	17 (8.1)	9 (10.2)	2 (5.0)	73 (37.8)	1.68 (0.67-4.25)	0.271	1.97 (0.54-7.18)	0.305	0.37 (0.07-2.03)	0.250
T	(+)	63 (30.1)	21 (23.9)	5 (12.5)	32 (16.6)	17.71 (6.87-45.66)*	<0.001	5.01 (1.40-17.88)*	0.013	1.87 (0.42-8.28)	0.411
G	(+)	120 (57.4)	53 (60.2)	28 (70.0)	31 (16.1)	38.89 (15.19-99.58)*	<0.001	18.07 (5.26-62.02)*	<0.001	12.72 (3.56-45.49)*	<0.001
rs1137070											
C		71 (34.0)	26 (29.6)	10 (25.0)	103 (53.4)	1.00 [Reference]		1.00 [Reference]		1.00 [Reference]	
T		138 (66.0)	62 (70.5)	30 (75.0)	90 (46.6)	2.16 (1.32-3.55)*	0.002	3.38 (1.75-6.52)*	<0.001	3.35 (1.47-7.62)*	0.004
C	(-)	9 (4.3)	5 (5.7)	5 (12.5)	70 (36.3)	1.00 [Reference]		1.00 [Reference]		1.00 [Reference]	
T	(-)	17 (8.1)	9 (10.2)	2 (5.0)	60 (31.1)	2.13 (0.84-5.36)	0.110	3.19 (0.89-11.50)	0.076	0.53 (0.10-2.89)	0.461
C	(+)	62 (29.7)	21 (23.9)	5 (12.5)	33 (17.1)	17.84 (7.16-44.45)*	<0.001	5.67 (1.69-19.07)*	0.005	2.23 (0.53-9.41)	0.275
T	(+)	121 (57.9)	53 (60.2)	28 (70.0)	30 (15.5)	44.38 (17.92-109.95)*	<0.001	22.45 (6.97-72.34)*	<0.001	15.42 (4.62-51.48)*	<0.001
rs5906957											
G		73 (34.9)	31 (35.2)	10 (25.0)	79 (40.9)	1.00 [Reference]		1.00 [Reference]		1.00 [Reference]	
A		136 (65.1)	57 (64.8)	30 (75.0)	114 (59.1)	1.60 (0.97-2.64)	0.068	1.93 (1.01-3.68)*	0.047	2.58 (1.12-5.93)*	0.025
G	(-)	9 (4.3)	6 (6.8)	5 (12.5)	48 (24.9)	1.0		1.0		1.0	
A	(-)	17 (8.1)	8 (9.1)	2 (5.0)	82 (42.5)	1.34 (0.52-3.40)	0.544	1.16 (0.33- 4.12)	0.822	0.27 (0.05-1.49)	0.133
G	(+)	64 (30.6)	25 (28.4)	5 (12.5)	31 (16.1)	15.54 (5.93-40.68)*	<0.001	4.24 (1.27- 14.24)*	0.019	1.53 (0.34-6.88)	0.578
A	(+)	119 (56.9)	49 (55.7)	28 (70.0)	32 (16.6)	32.50 (12.60-83.85)*	<0.001	11.70 (3.58- 38.19)*	<0.001	9.88 (2.76-35.40)*	<0.001
MAOB											
rs6324											
A		60 (28.7)	14 (15.9)	4 (10.0)	157 (81.4)	1.00 [Reference]		1.00 [Reference]		1.00 [Reference]	
G		149 (71.3)	74 (84.1)	36 (90.0)	36 (18.7)	11.32 (6.28-20.40)*	<0.001	20.48 (9.28-45.18)*	<0.001	38.04 (12.12-119.40)*	<0.001
A	(-)	5 (2.4)	0 (0.0)	1 (2.5)	108 (56.0)	1.00 [Reference]		1.00 [Reference]		1.00 [Reference]	
G	(-)	21 (10.1)	14 (15.9)	6 (15.0)	22 (11.4)	18.30 (6.02-55.70)*	<0.001	NA		30.50 (3.40-273.78)*	0.002
A	(+)	55 (26.3)	14 (15.9)	3 (7.5)	49 (25.4)	31.85 (10.79- 94.03)*	<0.001	NA		7.90 (0.74-84.76)	0.088

G	(+)	128 (61.2)	60 (68.2)	30 (75.0)	14 (7.3)	252.21 (78.89- 806.31)*	<0.001	NA		244.40 (27.74-NA)*	<0.001
rs1799836											
C		40 (19.1)	10 (11.4)	5 (12.5)	34 (17.6)	1.00 [Reference]		1.00 [Reference]		1.00 [Reference]	
T		169 (80.9)	78 (88.6)	35 (87.5)	159 (82.4)	1.14 (0.62-2.10)	0.678	2.53 (1.04-6.16)*	0.040	2.03 (0.70-5.92)	0.194
C	(-)	4 (1.9)	1 (1.1)	2 (5.0)	19 (9.8)	1.00 [Reference]		1.00 [Reference]		1.00 [Reference]	
T	(-)	22 (10.5)	13 (14.8)	5 (12.5)	111 (57.5)	1.17 (0.34-4.02)	0.808	2.78 (0.31-25.11)	0.364	0.44 (0.08-2.57)	0.362
C	(+)	36 (17.2)	9 (10.2)	3 (7.5)	15 (7.8)	19.57 (5.05-75.80)*	<0.001	7.41 (0.73-75.16)	0.090	1.84 (0.24-14.30)	0.562
T	(+)	147 (70.3)	65 (73.9)	30 (75.0)	48 (24.9)	23.90 (6.93-82.48)*	<0.001	20.25 (2.33-175.69)*	0.006	6.31 (1.20-33.11)*	0.029
rs3027452											
A		33 (15.8)	8 (9.1)	5 (12.5)	27 (14.0)	1.00 [Reference]		1.00 [Reference]		1.00 [Reference]	
G		176 (84.2)	80 (90.9)	35 (87.5)	166 (86.0)	1.17 (0.60-2.26)	0.646	2.59 (0.97-6.87)	0.057	1.61 (0.54-4.79)	0.390
A	(-)	3 (1.44)	1 (1.1)	2 (5.0)	13 (6.7)	1.00 [Reference]		1.00 [Reference]		1.00 [Reference]	
G	(-)	23 (11.0)	13 (14.8)	5 (12.5)	117 (60.6)	1.00 (0.24-4.09)	0.998	1.55 (0.16-14.98)	0.703	0.27 (0.04-1.60)	0.148
A	(+)	30 (14.4)	7 (8.0)	3 (7.5)	14 (7.3)	15.56 (3.37-71.92)*	<0.001	3.80 (0.34-43.13)	0.281	1.31 (0.16-10.73)	0.801
G	(+)	153 (73.2)	67 (76.1)	30 (75.0)	49 (25.4)	21.30 (5.18-87.57)*	<0.001	12.45 (1.33-116.49)*	0.027	3.99 (0.72-22.15)	0.113
COMT											
rs4633											
T/T		9 (4.3)	6 (6.8)	4 (10.0)	17 (8.8)	1.00 [Reference]		1.00 [Reference]		1.00 [Reference]	
C/T		65 (31.1)	26 (29.6)	7 (17.5)	81 (42.0)	1.30 (0.45-3.75)	0.625	0.60 (0.17-2.07)	0.415	0.30 (0.07-1.26)	0.099
C/C		135 (64.6)	56 (63.6)	29 (72.5)	95 (49.2)	2.27 (0.82-6.29)	0.115	1.08 (0.33-3.53)	0.898	1.05 (0.29-3.78)	0.946
T/T+C/T		74 (35.4)	32 (36.4)	11 (27.5)	98 (50.8)	1.00 [Reference]		1.00 [Reference]		1.00 [Reference]	
C/C		135 (64.6)	56 (63.6)	29 (72.5)	95 (49.2)	1.80 (1.10-2.95)*	0.019	1.65 (0.89-3.09)	0.115	2.63 (1.18-5.85)*	0.018
T/T+C/T	(-)	9 (4.3)	4 (4.6)	2 (5.0)	68 (35.2)	1.00 [Reference]		1.00 [Reference]		1.00 [Reference]	
C/C	(-)	17 (8.1)	10 (11.4)	5 (12.5)	62 (32.1)	1.76 (0.70-4.42)	0.231	1.83 (0.49-6.80)	0.370	2.56 (0.47-13.99)	0.278
T/T+C/T	(+)	65 (31.1)	28 (31.8)	9 (22.5)	30 (15.5)	19.66 (7.87-49.09)*	<0.001	7.66 (2.20-26.73)*	0.001	10.14 (1.83-56.06)*	0.008
CC	(+)	118 (56.5)	46 (52.3)	24 (60.0)	33 (17.1)	35.49 (14.37-87.64)*	<0.001	12.41 (3.65-42.19)*	<0.001	26.75 (5.16-138.55)*	<0.001
rs9605030											
C/C		130 (62.2)	47 (53.4)	16 (40.0)	115 (59.6)	1.00 [Reference]		1.00 [Reference]		1.00 [Reference]	
C/T		67 (32.1)	36 (40.9)	20 (50.0)	69 (35.8)	0.94 (0.56-1.58)	0.827	1.45 (0.76-2.76)	0.265	2.46 (1.12-5.40)*	0.025
T/T		12 (5.7)	5 (5.7)	4 (10.0)	9 (4.7)	1.02 (0.35-3.02)	0.972	1.21 (0.31-4.70)	0.778	3.32 (0.81-13.53)	0.094
C/C+C/T		197 (94.3)	83 (94.3)	36 (90.0)	184 (95.3)	1.00 [Reference]		1.00 [Reference]		1.00 [Reference]	
T/T		12 (5.7)	5 (5.7)	4 (10.0)	9 (4.7)	1.04 (0.36-3.04)	0.938	1.06 (0.28-4.02)	0.931	2.22 (0.58-8.51)*	0.244
C/C+C/T	(-)	26 (12.4)	14 (15.9)	5 (12.5)	123 (63.7)	1.00 [Reference]		1.00 [Reference]		1.00 [Reference]	
T/T	(-)	0 (0.0)	0 (0.0)	2 (5.0)	7 (3.6)	NA		NA		4.10 (0.59-28.42)	0.153
C/C+C/T	(+)	171 (81.8)	69 (78.4)	31 (77.5)	61 (31.6)	18.45 (9.63-35.36)*	<0.001	6.28 (2.80-14.08)*	<0.001	12.49 (3.89-40.02)*	<0.001
T/T	(+)	12 (5.7)	5 (5.7)	2 (5.0)	2 (1.0)	40.38 (7.53-216.62)*	<0.001	16.25 (2.35-112.37)*	0.005	32.03 (3.23-318.00)*	0.003
rs9606186											

C/C		17 (8.1)	5 (5.7)	3 (7.5)	23 (11.9)	1.00 [Reference]		1.00 [Reference]		1.00 [Reference]	
G/C		89 (42.6)	36 (40.9)	12 (30.0)	84 (43.5)	2.17 (0.94-5.00)	0.068	2.60 (0.78-8.66)	0.118	1.62 (0.39-6.77)	0.510
G/G		103 (49.3)	47 (53.4)	25 (62.5)	86 (44.6)	2.52 (1.11-5.75)*	0.028	3.74 (1.15-12.20)*	0.029	3.38 (0.86-13.29)	0.081
C/C+G/C		106 (50.7)	41 (46.6)	15 (37.5)	107 (55.4)	1.00 [Reference]		1.00 [Reference]		1.00 [Reference]	
G/G		103 (49.3)	47 (53.4)	25 (62.5)	86 (44.6)	1.36 (0.84-2.20)	0.214	1.72 (0.93-3.18)	0.082	2.32 (1.09-4.93)*	0.028
C/C+G/C	(-)	12 (5.7)	7 (8.0)	3 (7.5)	70 (36.3)	1.00 [Reference]		1.00 [Reference]		1.00 [Reference]	
G/G	(-)	14 (6.7)	7 (8.0)	4 (10.0)	60 (31.1)	1.39 (0.57-3.40)	0.469	1.79 (0.52-6.21)	0.359	1.52 (0.32-7.25)	0.597
C/C+G/C	(+)	94 (45.0)	34 (38.6)	12 (30.0)	37 (19.2)	20.50 (9.03-46.54)*	<0.001	7.13 (2.47-20.57)*	<0.001	7.95 (1.86-33.89)*	0.005
G/G	(+)	89 (42.6)	40 (45.5)	21 (52.5)	26 (13.5)	28.30 (11.92-67.20)*	<0.001	12.46 (4.24-36.59)*	<0.001	20.61 (4.89-86.94)*	<0.001

Abbreviations: AOR, adjusted odds ratio; BQ, betel-quid; OPMD, oral potentially malignant disorder; SNP, single nucleotide polymorphism; NA, not applicable owing to limited samples.

[a] May not total 100% due to rounding.

[b] AOR was obtained after adjustment for age, race, marital status, educational level, and covariates (alcohol, betel quid, and cigarette uses).

NA, non-appreciable owing to limited samples.

* $p < 0.05$

Table S4. The synergistic effects of cigarette smoking and susceptibility SNPs of MAO/COMT on oral and pharyngeal cancers and OPMD were calculated by stratifying the uses of cigarette across the susceptibility SNPs of MAO/COMT (N = 530).

		Oral and pharyngeal cancers (N = 297)	OPMD (N = 40)	Control (N = 193)	Oral and pharyngeal cancers vs. control		OPMD vs. control	
					Risk assessment		Risk assessment	
SNPs	CIG	N(%)[a]	N(%)	N(%)	AOR(95% CI)[b]	*p*	AOR(95% CI)[b]	*p*
MAOA								
rs6323								
T	(-)	14(4.7)	4(10.0)	23(11.9)	1.00[Reference]		1.00[Reference]	
G	(-)	22(7.4)	1(2.5)	20(10.4)	1.68(0.54-5.28)	0.374	0.28(0.03-2.86)	0.280
T	(+)	84(28.3)	6(15.0)	66(34.2)	0.71(0.26-1.95)	0.510	0.15(0.03-0.73)*	0.018
G	(+)	177(59.6)	29(72.5)	84(43.5)	1.51(0.57-3.97)	0.406	0.71(0.18-2.83)	0.626
rs1137070								
C	(-)	13(4.4)	4(10.0)	22(11.4)	1.00[Reference]		1.00[Reference]	
T	(-)	23(7.7)	1(2.5)	21(10.9)	1.97(0.62-6.21)	0.250	0.27(0.03-2.75)	0.268
C	(+)	84(28.3)	6(15.0)	81(42.0)	0.74(0.27-2.03)	0.560	0.15(0.03-0.71)*	0.016
T	(+)	177(59.6)	29(72.5)	69(35.8)	1.88(0.70-5.05)	0.210	0.83(0.21-3.28)	0.792
rs5906957								
G	(-)	17(5.7)	4(10.0)	19(9.8)	1.00[Reference]		1.00[Reference]	
A	(-)	19(6.4)	1(2.5)	24(12.4)	0.87(0.28-2.71)	0.811	0.18(0.02-1.90)	0.155
G	(+)	87(29.3)	6(15.0)	60(31.1)	0.50(0.18-1.33)	0.164	0.12(0.02-0.58)*	0.009
A	(+)	174(58.6)	29(72.5)	90(46.6)	0.98(0.39-2.51)	0.971	0.54(0.13-2.15)	0.380
MAOB								
rs6324								
A	(-)	11(3.7)	1(2.5)	33(17.1)	1.00[Reference]		1.00[Reference]	
G	(-)	25(8.4)	4(10.0)	10(5.2)	17.81(5.04-62.98)*	<0.001	22.12(2.04-239.66)*	0.011
A	(+)	63(21.2)	3(7.5)	124(64.3)	1.02(0.38-2.74)	0.973	0.40(0.04-4.30)	0.446
G	(+)	198(66.7)	32(80.0)	26(13.5)	12.39(4.48-34.21)*	<0.001	16.49(1.93-141.00)*	0.011
rs1799836								
C	(-)	6(2.0)	1(2.5)	5(2.6)	1.00[Reference]		1.00	
T	(-)	30(10.1)	4(10.0)	38(19.7)	0.53(0.11-2.50)	0.419	0.46(0.04-5.45)	0.537
C	(+)	44(14.8)	4(10.0)	29(15.0)	0.33(0.07-1.62)	0.170	0.16(0.01-2.09)	0.160
T	(+)	217(73.1)	31(77.5)	121(62.7)	0.53(0.12-2.37)	0.402	0.40(0.04-4.30)	0.451
rs3027452								
A	(-)	5(1.7)	1(2.5)	4(2.1)	1.00[Reference]		1.00[Reference]	
G	(-)	31(10.4)	4(10.0)	39(20.2)	0.59(0.10-3.34)	0.547	0.38(0.03-4.88)	0.460
A	(+)	36(12.1)	4(10.0)	23(11.9)	0.35(0.06-2.12)	0.253	0.16(0.01-2.31)	0.179
G	(+)	225(75.8)	31(77.5)	127(65.8)	0.56(0.10-3.02)	0.497	0.32(0.03-3.68)	0.363
COMT								
rs4633								
T/T+C/T	(-)	11(3.7)	1(2.5)	23(11.9)	1.00[Reference]		1.00[Reference]	
C/C	(-)	25(8.4)	4(10.0)	20(10.4)	3.07(0.93-10.13)	0.065	5.48(0.53-56.65)	0.153
T/T+C/T	(+)	95(32.0)	10(25.0)	75(38.9)	1.29(0.44-3.76)	0.641	1.26(0.13-11.71)	0.842
C/C	(+)	166(55.9)	25(62.5)	75(38.9)	2.06(0.72-5.90)	0.177	2.90(0.33-25.64)	0.338
rs9605030								
C/C+C/T	(-)	33(11.1)	4(10.0)	41(21.2)	1.00[Reference]		1.00[Reference]	
T/T	(-)	3(1.0)	1(2.5)	2(1.0)	0.41(0.04-4.24)	0.454	1.65(0.09-29.75)	0.736
C/C+C/T	(+)	247(83.2)	32(80.0)	143(74.1)	0.78(0.39-1.60)	0.502	0.72(0.20-2.58)	0.614
T/T	(+)	14(4.7)	3(7.5)	7(3.6)	1.02(0.28-3.75)*	0.977	1.72(0.27-11.15)	0.570
rs9606186								
C/C+G/C	(-)	16(5.4)	3(7.5)	24(12.4)	1.00[Reference]		1.00[Reference]	
G/G	(-)	20(6.7)	2(5.0)	19(9.8)	2.46(0.80-7.58)	0.117	1.22(0.17-8.63)	0.841

C/C+G/C (+)	131(44.1)	12(30.0)	83(43.0)	1.16(0.46-2.89)	0.757	0.47(0.11-2.08)	0.318
G/G (+)	130(43.8)	23(57.5)	67(34.7)	1.48(0.59-3.71)*	0.408	1.15(0.27-4.84)	0.849

Abbreviations: AOR, adjusted odds ratio; CIG, cigarette; OPMD, oral potentially malignant disorder; SNP, single nucleotide polymorphism; NA, non-appreciable owing to limited samples. [a] May not total 100% due to rounding. [b] AOR was obtained after adjustment for age, race, marital status, educational level, and covariates (alcohol, and BQ). * $p < 0.05$.

Table S5. The synergistic effects of alcohol drinking and susceptibility SNPs of MAO/COMT on oral and pharyngeal cancers and OPMD were calculated by stratifying the uses of alcohol across the susceptibility SNPs of MAO/COMT (N = 530).

	Oral and pharyngeal cancers (N = 297)	OPMD (N = 40)	Control (N = 193)	Oral and pharyngeal cancers vs. control		OPMD vs. control	
				Risk assessment		Risk assessment	
SNPs ALC	N(%)[a]	N(%)	N(%)	AOR(95% CI)[b]	*p*	AOR(95% CI)[b]	*p*
MAOA							
rs6323							
T (-)	26(8.8)	4(10.0)	47(24.4)	1.00[Reference]		1.00[Reference]	
G (-)	45(15.2)	9(22.5)	49(25.4)	1.92(0.86-4.28)	0.109	2.19(0.58-8.21)	0.246
T (+)	72(24.2)	6(15.0)	42(21.8)	1.30(0.58-2.87)	0.525	0.56(0.13-2.42)	0.441
G (+)	154(51.9)	21(52.5)	55(28.5)	2.64(1.26-5.54)*	0.010	1.91(0.55-6.73)	0.312
rs1137070							
C (-)	26(8.8)	4(10.0)	44(22.8)	1.00[Reference]		1.00[Reference]	
T (-)	45(15.2)	9(22.5)	52(26.9)	1.24(0.56-2.75)	0.600	1.49(0.40-5.57)	0.554
C (+)	71(23.9)	6(15.0)	59(30.6)	0.82(0.37-1.81)	0.626	0.39(0.09-1.66)	0.202
T (+)	155(52.2)	21(52.5)	38(19.7)	2.81(1.29-6.14)*	0.009	2.09(0.58-7.57)	0.262
rs5906957							
G (-)	25(8.4)	3(7.5)	39(20.2)	1.00[Reference]		1.00[Reference]	
A (-)	46(15.5)	10(25.0)	57(29.5)	1.56(0.69-3.53)	0.289	2.25(0.54-9.45)	0.268
G (+)	79(26.6)	7(17.5)	40(20.7)	1.32(0.58-3.01)	0.507	0.73(0.16-3.44)	0.695
A (+)	147(49.5)	20(50.0)	57(29.5)	2.30(1.05-5.04)*	0.037	2.01(0.49-8.16)	0.331
MAOB							
rs6324							
A (-)	20(6.7)	1(2.5)	78(40.4)	1.00[Reference]		1.00[Reference]	
G (-)	51(17.2)	12(30.0)	18(9.3)	9.42(3.80-23.38)*	<0.001	51.13(5.86-445.81)*	<0.001
A (+)	54(18.2)	3(7.5)	79(40.9)	1.01(0.47-2.16)	0.988	1.18(0.12-12.25)	0.887
G (+)	172(57.9)	24(60.0)	18(9.3)	15.54(6.81-35.45)*	<0.001	39.98(4.80-332.98)*	<0.001
rs1799836							
C (-)	10(3.4)	3(7.5)	14(7.3)	1.00[Reference]		1.00	
T (-)	61(20.5)	10(25.0)	82(42.5)	1.20(0.41-3.47)	0.741	0.57(0.13-2.58)	0.465
C (+)	40(13.5)	2(5.0)	20(10.4)	1.16(0.35-3.81)	0.810	0.14(0.02-1.08)	0.059
T (+)	186(62.6)	25(62.5)	77(39.9)	1.76(0.62-5.02)	0.293	0.63(0.14-2.75)	0.537
rs3027452							
A (-)	8(2.7)	3(7.5)	12(6.2)	1.00[Reference]		1.00[Reference]	
G (-)	63(21.2)	10(25.0)	84(43.5)	1.25(0.39-3.98)	0.706	0.48(0.10-2.25)	0.353
A (+)	33(11.1)	2(5.0)	15(7.8)	1.19(0.32-4.38)	0.798	0.14(0.02-1.17)	0.070
G (+)	193(65.0)	25(62.5)	82(42.5)	1.81(0.58-5.66)	0.309	0.51(0.11-2.30)	0.383
COMT							
rs4633							
T/T+C/T (-)	30(10.1)	4(10.0)	49(25.4)	1.00[Reference]		1.00[Reference]	
C/C (-)	41(13.8)	9(22.5)	47(24.4)	1.82(0.83-4.03)	0.137	2.72(0.73-10.20)	0.137
T/T+C/T (+)	76(25.6)	7(17.5)	49(25.4)	1.40(0.65-3.00)	0.392	0.79(0.20-3.20)	0.742
C/C (+)	150(50.5)	20(50.0)	48(24.9)	2.45(1.19-5.07)*	0.015	2.05(0.59-7.14)	0.258
rs9605030							
C/C+C/T (-)	67(22.6)	12(30.0)	92(47.7)	1.00[Reference]		1.00[Reference]	
T/T (-)	4(1.4)	1(2.5)	4(2.1)	0.72(0.11-4.73)	0.734	1.11(0.09-14.11)	0.938
C/C+C/T (+)	213(71.7)	24(60.0)	92(47.7)	1.36(0.79-2.35)	0.261	0.75(0.31-1.81)	0.521
T/T (+)	13(4.4)	3(7.5)	5(2.6)	1.69(0.47-6.12)	0.424	2.29(0.43-12.33)	0.334
rs9606186							
C/C+G/C (-)	37(12.5)	7(17.5)	49(25.4)	1.00[Reference]		1.00[Reference]	
G/G (-)	34(11.5)	6(15.0)	47(24.4)	1.56(0.71-3.42)	0.270	1.24(0.36-4.32)	0.732

C/C+G/C	(+)	110(37.0)	8(20.0)	58(30.1)	1.51(0.75-3.03)	0.245	0.48(0.15-1.57)	0.226
G/G	(+)	116(39.1)	19(47.5)	39(20.2)	2.09(1.01-4.30)*	0.046	1.52(0.52-4.50)	0.448

Abbreviations: AOR, adjusted odds ratio; ALC, alcohol; OPMD, oral potentially malignant disorder; SNP, single nucleotide polymorphism; NA, non-appreciable owing to limited samples. [a] May not total 100% due to rounding. [b] AOR was obtained after adjustment for age, race, marital status, educational level, and covariates (BQ, and cigarette uses). * $p < 0.05$.

Table S6. Distribution of significantly demographic characteristics and substance use among males with oral and pharyngeal cancers and control were calculated after propensity-score matching for age (N = 300).

Demography and substance use factors	After age matching: Oral and pharyngeal cancers (N = 150) Mean ± SD N(%)[a]	Control (N = 150) Mean ± S.D N(%)	p
**Age, years (mean ± SD)**	50.31 ± 11.00†	49.61 ± 11.71	0.598*[b]
Age group ≤ 50 (years)	85(56.7)	85(56.7)	1.000*[c]
Age group > 50 (years)	65(43.3)	65(43.3)	
**Ethnicity**			
Minnan	107(71.3)	123(82.0)	0.029*[c]
Non-Minnan	43(28.7)	27(18.0)	
**Marital status**			
Unmarried	23(15.3)	9(6.0)	0.009*[c]
Married	127(84.7)	141(94.0)	
**Education level**			
Low (≤ 9 years)	91(60.7)	62(41.3)	0.001*[c]
High (> 9 years)	59(39.3)	88(58.7)	
**Alcohol drinking status**			
Never	39(26.0)	74(49.3)	<0.001*[c]
Current or former	111(74.0)	76(50.7)	
**BQ chewing status**			
Never	21(14.0)	102(68.0)	<0.001*[c]
Current or former	129(86.0)	48(32.0)	
**Cigarette smoking status**			
Never	18(12.0)	39(26.0)	0.002*[c]
Current or former	132(88.0)	111(74.0)	

Abbreviations: BQ, betel-quid. [a] May not total 100% due to rounding. [b] Significant difference was test by independent *t-test* (* $p < 0.05$). [c] Significant difference was test by Chi-square analysis (* $p < 0.05$). SD: standard deviation; BQ: betel quid.

Table S7. The susceptibility SNPs of MAO/COMT on oral and pharyngeal cancers were calculated after propensity-score matching for age (N = 300).

	**Oral and pharyngeal cancers (N = 150)**	**Control (N = 150)**	**After age matching**	
			**Oral and pharyngeal cancers vs. control**	
			Risk assessment	
SNPs	N(%)[a]	N(%)	AOR(95% CI)[b]	*p*
MAOA				
rs6323				
T	48(32.0)	70(46.7)	1.00[Reference]	
G	102(68.0)	80(53.3)	2.13(1.19-3.80)*	0.011
rs1137070				
C	47(31.3)	76(50.7)	1.00[Reference]	
T	103(68.7)	74(49.3)	2.24(1.25-3.99)*	0.006
rs5906957				
G	53(35.3)	62(41.3)	1.00[Reference]	
A	97(64.7)	88(58.7)	1.36(0.77-2.43)	0.292
MAOB				
rs6324				
A	33(22.0)	122(81.3)	1.00[Reference]	
G	117(78.0)	28(18.7)	15.54(7.70-31.38)*	<0.001
rs1799836				
C	27(18.0)	26(17.3)	1.00[Reference]	
T	123(82.0)	124(82.7)	0.96(0.47-1.99)	0.919
rs3027452				
A	23(15.3)	20(13.3)	1.00[Reference]	
G	127(84.7)	130(86.7)	1.05(0.48-2.27)	0.911
COMT				
rs4633				
T/T	9(6.0)	16(10.7)	1.00[Reference]	
C/T	42(28.0)	62(41.3)	0.91(0.30-2.78)	0.863
C/C	99(66.0)	72(48.0)	1.96(0.68-5.68)	0.217
T/T+C/T	51(34.0)	78(52.0)	1.00[Reference]	
C/C	99(66.0)	72(48.0)	2.12(1.20-3.76)*	0.010
rs9605030				
C/C	99(66.0)	87(58.0)	1.00[Reference]	
C/T	44(29.3)	55(36.7)	0.83(0.45-1.51)	0.535
T/T	7(4.7)	8(5.3)	0.99(0.27-3.68)	0.991
C/C+C/T	143(95.3)	142(94.7)	1.00[Reference]	
T/T	7(4.7)	8(5.3)	1.06(0.29-3.87)	0.932
rs9606186				
C/C	12(8.0)	19(12.7)	1.00[Reference]	
G/C	61(40.7)	66(44.0)	2.08(0.81-5.32)	0.128
G/G	77(51.3)	65(43.3)	2.99(1.17-7.62)*	0.022
C/C+G/C	73(48.7)	85(56.7)	1.00[Reference]	
G/G	77(51.3)	65(43.3)	1.68(0.96-2.94)	0.072

Abbreviations: AOR, adjusted odds ratio; BQ, betel-quid; OPMD, oral potentially malignant disorder; SNP, single nucleotide polymorphism; NA, non-applicable owing to limited samples. [a] May not total 100% due to rounding. [b] AOR was obtained after adjustment for age, race, marital status, educational level, and covariates (alcohol, betel quid, and cigarette uses). * $p <$ 0.05.

Table S8. Distribution of selected clinical characteristics in males with oral and pharyngeal cancers (N = 42).

Variables	Oral site (N = 28)	Pharynx site (N = 14)		
	N (%)	N (%)	Chi-square *p*	Mann- Whitney U *p*
**Clinical characteristics**				
TNM Stage				
II+III	16 (57.1)	8 (57.1)	1.000	
IV	12 (42.9)	6 (42.9)		
Age (years), Mean (SD)	54.64 (10.34)	56.57 (7.35)	0.470	
BMI (kg/m^2), Mean (SD)	25.55 (3.85)	22.47 (4.83)		0.111
Race				
Minnan	23 (82.1)	10 (71.4)	0.425	
Non-Minnan	5 (17.9)	4 (28.6)		
Marital status				
Married	17 (60.7)	6 (42.9)		
Unmarried	11 (39.3)	8 (57.1)	0.273	
Occupational status				
Working	16 (57.1)	5 (35.7)	0.190	
Not working	12 (42.9)	9 (64.3)		
Education level				
Low (≤ 6 year)	19 (67.9)	11 (78.6)	0.469	
High (> 6 year)	9 (32.1)	3 (21.4)		
Religion status				
Having a religion	24 (85.7)	11 (78.6)	0.558	
No religion	4 (14.3)	3 (21.4)		
Alcohol drinking status				
Never	8 (28.6)	2 (14.3)	0.306	
Current or former	20 (71.4)	12 (85.7)		
Age at starting drinking (years), Mean (SD)	21.53 (6.49)	20.44 (3.81)		0.620
Years of alcohol drinking, Mean (SD)	30.87 (10.39)	34.11 (11.66)		0.389
BQ chewing status				
Never	2 (7.1)	3 (21.4)	0.178	
Current or former	26 (92.9)	11 (78.6)		
Age at starting chewing (years), Mean (SD)	22.80 (5.80)	21.78 (4.87)		0.692
Years of BQ chewing, Mean (SD)	24.78 (10.45)	32.00 (12.33)		0.061
Average amount of chewing, Mean (SD)	31.13 (26.52)	23.89 (8.58)		0.903
Cumulative lifetime BQ use (pack-years), Mean (SD)	78.86 (74.20)	71.83 (32.46)		0.686
Type of BQ material				
With Piper betel leaf	17 (60.7)	4 (28.6)		
Mixed use (With Piper betel leaf or with inflorescence of Piper betel Linn.)	11 (39.3)	10 (71.4)	0.050	
Cigarette smoking status				
Never	2 (7.1)	1 (7.1)	1.000	
Current or former	26 (92.9)	13 (92.9)		
Age at starting smoking (years), Mean (SD)	19.72 (4.70)	19.20 (3.36)		0.554
Years of cigarette smoking, Mean (SD)	31.84 (9.04)	35.30 (10.86)		0.257
Average amount of smoking, Mean (SD)	12.86 (2.57)	17.00 (5.38)		0.758

BMI: body mass index
SD: standard deviation
BQ: betel quid

Table S9. Associations between MAOA, MAOB, and COMT mRNA expression (N = 42).

Biomarkers	Spearman's Rho correlation coefficient	*p*
MAOA-MAOB	0.822*	<0.001
MAOB-COMT	0.738*	<0.001
MAOA-COMT	0.557*	<0.001

* $p < 0.05$
